# A Natural Light/Dark Cycle Regulation of Carbon-Nitrogen Metabolism and Gene Expression in Rice Shoots

**DOI:** 10.3389/fpls.2016.01318

**Published:** 2016-08-30

**Authors:** Haixing Li, Zhijun Liang, Guangda Ding, Lei Shi, Fangsen Xu, Hongmei Cai

**Affiliations:** Plant Nutrigenic and Molecular Biology, College of Resources and Environment, Huazhong Agricultural UniversityWuhan, China

**Keywords:** carbon, nitrogen, metabolism, gene, miRNA

## Abstract

Light and temperature are two particularly important environmental cues for plant survival. Carbon and nitrogen are two essential macronutrients required for plant growth and development, and cellular carbon and nitrogen metabolism must be tightly coordinated. In order to understand how the natural light/dark cycle regulates carbon and nitrogen metabolism in rice plants, we analyzed the photosynthesis, key carbon-nitrogen metabolites, and enzyme activities, and differentially expressed genes and miRNAs involved in the carbon and nitrogen metabolic pathway in rice shoots at the following times: 2:00, 6:00, 10:00, 14:00, 18:00, and 22:00. Our results indicated that more CO_2_ was fixed into carbohydrates by a high net photosynthetic rate, respiratory rate, and stomatal conductance in the daytime. Although high levels of the nitrate reductase activity, free ammonium and carbohydrates were exhibited in the daytime, the protein synthesis was not significantly facilitated by the light and temperature. In mRNA sequencing, the carbon and nitrogen metabolism-related differentially expressed genes were obtained, which could be divided into eight groups: photosynthesis, TCA cycle, sugar transport, sugar metabolism, nitrogen transport, nitrogen reduction, amino acid metabolism, and nitrogen regulation. Additionally, a total of 78,306 alternative splicing events have been identified, which primarily belong to alternative 5′ donor sites, alternative 3′ acceptor sites, intron retention, and exon skipping. In sRNA sequencing, four carbon and nitrogen metabolism-related miRNAs (osa-miR1440b, osa-miR2876-5p, osa-miR1877 and osa-miR5799) were determined to be regulated by natural light/dark cycle. The expression level analysis showed that the four carbon and nitrogen metabolism-related miRNAs negatively regulated their target genes. These results may provide a good strategy to study how natural light/dark cycle regulates carbon and nitrogen metabolism to ensure plant growth and development.

## Introduction

Nitrogen is one of the essential macronutrients required for plant growth and development. Nitrogen is not only a constituent of key cell molecules, such as nucleic acids, ATP, amino acids, chlorophyll, and several plant hormones, but is also the pivotal regulator of numerous biological processes, including amino acid metabolism, protein synthesis, and carbon metabolism (Frink et al., 1999; Crawford and Forde, 2002). Therefore, nitrogen is often a major limiting factor for plant productivity and crop yield (Lam et al., 1996; Tabuchi et al., 2007). Carbon plays a crucial role in routine plant growth and development. Various carbon compounds, including organic acids, sucrose, glucose, and other carbohydrates, provide both the energy and the carbon skeletons for ammonium (NH${}_{4}^{+}$) assimilation. Nitrogen compounds are synthesized by incorporating NH${}_{4}^{+}$ into the carbon skeletons to produce various amino acids and subsequently proteins. Amino acids, proteins, and particularly enzymes, are essential for almost all cellular activities, including the nitrogen and carbon metabolic reactions (Zheng, 2009). Therefore, both nitrogen and carbon nutrients are essential for plant cellular functions. Recently, it has been recognized that cellular carbon and nitrogen metabolism must be tightly coordinated (Zheng, 2009; Nunes-Nesi et al., 2010; Bao et al., 2014, 2015b). Maintaining a coordination of carbon-nitrogen metabolism and an appropriate balance of carbohydrates to nitrogen metabolites, also referred to as the “carbon/nitrogen balance,” is important for plant growth, development, and yield production (Coruzzi and Zhou, 2001; Krapp et al., 2002; Martin et al., 2002; Krapp and Truong, 2005; Zheng, 2009; Nunes-Nesi et al., 2010). Our previous study also indicated the importance of carbon-nitrogen metabolic balance in rice growth and development (Bao et al., 2014, 2015a,b).

The Earth's rotation around its axis generates daily and seasonal light and temperature changes. In order to coordinate these periodic environmental changes, many organisms have acquired the capacity to regulate their critical biological processes temporally by an endogenous rhythm, known as the circadian clock (Dodd et al., 2005; Harmer, 2009). Because plants are sessile, light, and temperature are two particularly important environmental cues for plant survival, as they must be able to sense environmental changes and adapt to them properly from day to day and season to season. The plant circadian clock generates a light/dark cycle close to 24 h. During the daytime, photosynthesis provides the plant energy and carbohydrates for growth and development, while at nighttime, plant metabolism, and growth depend on the carbohydrates that accumulated from photosynthesis during the day time to avoid starvation in the dark. A previous study reported that approximately 50% of the carbon assimilated during the daytime accumulates as starch in the leaves of *Arabidopsis thaliana* (Zeeman and Rees, 1999). At night, starch is degraded almost linearly to provide sugars for plant growth, with 5–10% remaining at dawn (Gibon et al., 2004; Smith and Stitt, 2007; Graf and Smith, 2011).

Therefore, to ensure the optimal physiology, growth, and behavior in light/dark cycles, a wide variety of plant metabolic events are regulated by the circadian clock and show the daily oscillation patterns, including photosynthesis, nutrient assimilation, redox homeostasis, starch metabolism, and secondary metabolism (Dodd et al., 2005; Lu et al., 2005; Gutiérrez et al., 2008; Graf et al., 2010; Kerwin et al., 2011; Lai et al., 2012). Metabolomics analysis revealed that ~30% of the measured primary metabolites showed the circadian clock controlled in *Arabidopsis* (Espinoza et al., 2010). Additionally, various transcriptional and post-transcriptional events are regulated by the circadian clock, which changes the plants' responses and sensitivities to various external stimuli and makes them obtain specific information throughout the day and night. Transcriptome analysis revealed that about one third of all genes are circadian clock regulated in *Arabidopsis*, such as those involved in photosynthesis and starch metabolism, nitrogen and sulfur metabolism, chlorophyll and carotene biosynthesis, flavonoid and anthocyanin biosynthesis, sugar membrane transportation, and redox balance (Covington et al., 2008). Sato et al. (2013) also released the diurnal and circadian gene expression profile of rice leaf throughout entire growth in the field during sunrise, mid-day (12:00), sunset, and midnight (24:00) in RiceXPro. Post-transcriptional control, mediated largely through alternative splicing and miRNAs, has also been shown to have a rapid response to the circadian clock and environmental changes (Bartok et al., 2013). The first step of pre-mRNA splicing is the recognition of a specific target sequence in the intron-exon junction or inside the intron. If the splicing acceptor site does not perfectly fit the consensus sequence of the region, an alternative version of mRNA can be generated (Cáceres and Kornblihtt, 2002). This process is called alternative splicing and can lead to the generation of many mRNA isoforms from a single genomic sequence. A recent study demonstrated the importance of alternative splicing in *Arabidopsis* mediating circadian responses to temperature changes using a genome-wide approach (James et al., 2012). MiRNA is one of the post-transcriptional regulators of gene expression; it completely or partially binds with the target mRNA and mainly reduces the translation level or accelerates the turnover of target mRNA. MiRNAs have also been implicated in both the core circadian mechanism, and in the responses of the clock to the environment, especially in *Drosophila* and mammals (Bartok et al., 2013).

To date, studies have focused on the circadian regulation of photosynthesis, carbon metabolism, and allocation in plants under controlled conditions. Few studies have reported on the light/dark cycle regulation of plant nitrogen metabolism under natural field conditions. Moreover, there is still no comprehensive, whole-genome RNAseq, and small RNAseq analysis to study the transcriptional and post-transcriptional regulation of the natural light/dark cycle in rice plants. There is a close interaction between the carbon and nitrogen metabolism in plants. Thus, in this study, we first analyze the photosynthesis, key carbon-nitrogen metabolites, and enzyme activities in rice shoots at the times: 2:00, 6:00, 10:00, 14:00, 18:00, and 22:00, to understand how the natural light/dark cycle regulates carbon and nitrogen metabolism in rice plants. Later, we analyze the transcriptome and small RNAs in rice shoots at each of the time points mentioned above, using mRNAseq and sRNAseq techniques, to reveal the differentially expressed genes and miRNAs involving in the carbon and nitrogen metabolic pathway. The results demonstrated that in rice shoots, not only were metabolites and enzyme activities regulated by the natural light/dark cycle but also genes, miRNAs and alternative splicing events.

## Materials and methods

### Plant growth and sample harvesting

The seeds of Zhonghua 11 (*Oryza sativa* ssp. *Japonica*) were germinated and sowed in sand at the potting farm at Huazhong Agricultural University, Wuhan, China. At the five-leaf stage, the seedlings were planted in the field. After 4 weeks, three biological replicated fresh samples of the rice shoots were harvested at 2:00, 6:00, 10:00, 14:00, 18:00, and 22:00. Each sample contained three plants. All samples were stored at −70°C and used for further analysis. At the same time, we monitored the photosynthetic parameters and the temperature when we harvested the samples at each time point. In the mRNA and small RNA sequencing analysis, we used 2:00 as the control time point to analyze the differentially expressed genes and miRNAs, because there was a lowest net photosynthetic rate in leaves at that time. Sunrise was at 5:00 and sundown at 19:00. Therefore, the daytime was from 5:00 to 19:00. Supplementary Figure S1 shows the diagram of rice germination, planting, and harvesting in this study.

### Carbon-nitrogen metabolites determination

- Photosynthetic parameter determination. At each time point, 10 plants were randomly selected for the determination of the photosynthetic parameters. The net photosynthetic rate, stomatal conductance, intercellular CO_2_ concentration, and transpiration rate of the first and second leaves from the top of the rice plants were tested by a Li-6400XT portable photosynthesis system (USA, Li-COR).
- Chlorophyll content determination. Chlorophyll a and b contents were estimated according to the procedure described by Porra et al. (1989). Approximately 0.2 g shoot samples were excised into pieces and homogenized in 80% (v/v) acetone and kept in a dark room at 4°C until the green samples turned white. After centrifuging, the absorbency of the extracted solution was measured at 662 and 645 nm by a spectrophotometer. The chlorophyll content was calculated according to the formula given by Zhang (1990).
- Free ammonium determination. Samples were freshly ground and homogenized with the extraction buffer [50 mM Tris HCl (pH 7.0), 10 mM imidazole, and 0.5% (w/v) β-mercaptoethanol] on ice. The homogenates were centrifuged at 12,000 g for 20 min at 4°C. Free ammonium in the supernatant was determined by the Berthelot color reaction method (Hausler et al., 1994). The absorbance of the reacted solution was measured at 480 nm by a spectrophotometer, and the free ammonium content was calculated from the standard curve of NH_4_NO_3_.
- Free amino acid determination. All steps followed the manufacturer's instructions (Hitachi Instruments Engineering, Japan). Samples were excised into pieces and immersed immediately in 80% (w/v) ethanol at 80°C three times. Free amino acids were extracted and underwent diethylether extraction, freeze drying, and dissolution in 0.2 mM HCl. Finally, the samples were analyzed by a Hitachi amino acid analyzer (L-8800) using leucine as a standard.
- Soluble protein and carbohydrate determination. For soluble protein analysis, samples were freshly ground and homogenized with the extraction buffer [10 mM Trizma (pH 7.5), 10 mM MgSO_4_, 5 mM sodium glutamate, 1 mM dithiothreitol, 10% (v/v) glycerol, and 0.05% (v/v) Triton X-100] on ice. The homogenates were centrifuged at 12,000 g for 20 min at 4°C (Melo et al., 2003). The soluble protein concentration in the supernatant was measured according to the Bradford method (Bradford, 1976) using Coomassie Plus Protein Assay Reagent (Pierce, Rockford, IL, USA). Bovine serum albumin was used as the standard protein. The soluble carbohydrates were extracted from pre-dried samples with boiling water and colorimetrically measured according to the Anthrone procedure (Morris, 1948; Maness, 2010).

### Carbon-nitrogen metabolic enzyme activity measurement

- Nitrate reductase activity measurement. Nitrate reductase activity in each sample was estimated based on the method described by Nakagawa et al. (1985). Approximately 0.5 g shoot samples were freshly ground and homogenized with the extraction buffer [0.05 g PVP; 0.5% (w/v) PEG; 0.25% (w/v) Na_2_S_2_O_3_ and 15% (v/v) glycerol] on ice. The homogenates were centrifuged at 12,000 g for 20 min at 4°C. The determination of nitrate reductase activity in the supernatant was based on the formation of nitrite in a reaction medium containing 0.1 ml enzyme extract, 0.2 ml 13 mM NADH, and 1.2 ml buffer. The reaction was terminated by adding 1 ml 1% sulfanilamide and 1 ml 0.02% naphthyl ethylenediamine-HCl.
- Glutamine synthetase activity measurement. Samples were freshly ground with the extraction buffer [70 mM MOPS (pH 6.8), 10 mM MgSO_4_, 5 mM glutamate, 2 mM dithiothreitol, 10% (v/v) ethanediol and 0.1% (v/v) Triton X-100] on ice. The homogenates were centrifuged at 12,000 g for 30 min at 4°C and the supernatant was used for glutamine synthetase activity analysis, according to the procedure described by Harrison et al. (2003). The supernatant was assayed with a pre-incubated buffer [100 mM glutamate, 70 mM MOPS (pH 6.80), 50 mM MgSO_4_, 15 mM NH_2_OH, and 15 mM ATP] at 37°C. The reaction was terminated after 30 min at 37°C by adding acidic FeCl_3_ solution (670 mM HCl, 200 mM trichloroacetic acid and 88 mM FeCl_3_). After allowing 10 min for the color to develop, the reaction mixture was centrifuged at 4000 g at room temperature for 10 min. The absorbency of the supernatant was measured at 540 nm by a spectrophotometer, and the glutamine synthetase activity was calculated by quantifying the formation of γ-glutamylhydroxamate (γ-GHA).
- Glutamate synthase activity measurement. Approximately 0.2 g samples were freshly ground and homogenized with 1.5 ml extraction buffer [50 mM Tris HCl (pH 7.5), 10 mM MgCl_2_, 1 mM EGTA, 1 mM EDTA,1 mM benzamidine, 1 mM PMSF, 1 mM ε-aminocaproic acid and 10 μM leupeptin] on ice. The homogenates were centrifuged at 12,000 g for 20 min at 4°C, and the supernatant was used for NADH- and Fd-glutamate synthase activity analysis, according to the procedure described by Hecht et al. (1988) and Migge et al. (1997), respectively.
- Glutamate dehydrogenase activity measurement. Approximately 0.5 g samples were freshly ground and homogenized with 1.5 ml extraction buffer [50 mM Tris HCl (pH 8.0), 1 mM DTT, 1 mM MgCl_2_, 1.0 mM MnCl_2_] on ice. The homogenates were centrifuged at 10,000 g for 45 min at 4°C, and the supernatant was used for glutamate dehydrogenase activity measurement, according to the procedure described by Kates and Jones (1964). Then, 0.1 ml supernatant was reacted with 3 ml reaction mixture [100 μM NH_4_Cl, 33 μM Tris HCl (pH 9.0), 4.0 μM α-ketoglutarate (pH 7.0), 1.0 μM CaCl_2_, 1.0 μM MgCl_2_, 0.3 μM NADH, 0.1 μM DTT] at 37°C for 20 min. The absorbency of the reaction medium was measured at 340 nm by a spectrophotometer, and the glutamate dehydrogenase activity was calculated by quantifying the decreasing of NADH.

### cDNA library construction and sequencing

For mRNA and small RNA sequencing analysis, the RNA extraction, cDNA library construction, and sequencing of rice shoot samples were carried out as a custom service by Personalbio (http://www.personalbio.cn). Total RNA was extracted by Trizol reagent (Invitrogen, Germany), according to the manufacturer's instructions. The cDNA library was constructed by TruSeq RNA Sample Prep Kit (Illumina), quantified by Quantifluor-ST fluorometer (Promega) and Quant-iTPicoGreen dsDNA Assay Kit (Invitrogen), qualified by Agilent 2100 Bioanalyzer and Agilent High Sensitivity DNA Kit. Later, samples were sequenced by Illumina Hiseq 2000.

### Data analysis

The original data quality evaluation, mapping back to the reference transcriptome gene, and small RNA annotation and expression analysis, and other bioinformatics analysis were also performed as a custom service by Personalbio (http://www.personalbio.cn). For the data from the mRNA sequencing, the useful reads were obtained by removing the ligation sequence and low quality sequences from original reads, and mapping to the rice reference genome (Oryza_sativa.IRGSP-1.0.21; ftp://ftp.ensemblgenomes.org/pub/release-21/plants/genbank/oryza_sativa) by Bowtie2/tophat2 (http://tophat.cbcb.umd.edu). Genes were annotated from Oryza_sativa.IRGSP-1.0.21.gtf (ftp://ftp.ensemblgenomes.org/pub/release-21/plants/gtf/oryza_sativa), and categorized by their biological functions from eggNOG analysis (http://www.ncbi.nlm.nih.gov/COG, http://eggnog.embl.de/version_3.0; Tatusov et al., 2003; Powell et al., 2011). For the data from the small RNA sequencing, the clean reads (15–30 nt) were obtained by removing the ligation sequence and low quality sequences from raw reads. The unique sequences were mapped to the rice reference genome by Bowtie. Small RNAs were annotated by blasting with the database of Rfam (11.0), and miRNAs were blasted with the mature miRNA sequences in rice. The number of mapped genes was analyzed by HTseq (http://www-huber.embl.de/users/anders/HTSeq), and the differentially expressed genes were analyzed by DEseq (http://www-huber.embl.de/users/anders/DESeq) with parameters *p* < 0.05, fold change > 2 and FDR < 0.001. The metabolic pathways of differentially expressed genes were analyzed by KEGG orthology (http://www.genome.jp/kegg/tool/map_pathway2.html; Kanehisa et al., 2004). The differentially expressed genes and miRNAs were clustered by Cluster 3.0 /TreeView (http://bonsai.hgc.jp/~mdehoon/software/cluster/software.htm, http://jtreeview.sourceforge.net). The target genes of miRNAs were predicted by psRNATarget (http://plantgrn.noble.org/psRNATarget/?function=3).

### QRT-PCR analysis

For qRT-PCR analysis, the total RNA was extracted with TriZol reagent (Invitrogen, Germany) and the first-strand cDNAs were synthesized from DNaseI-treated total RNA using Superscript II reverse transcriptase (Invitrogen, Germany) according to the manufacturer's instructions. QRT-PCR was performed in an optical 96-well-plate with an ABI PRISM 7500 real-time PCR system (Applied Biosystems, Foster City, CA, USA). Each reaction contained 12.5 μl of 2 × SYBR Green Master Mix reagent (Applied Biosystems), 3.0 μl of cDNA, and 200 μM each of the gene-specific primers in a final volume of 25 μl. The thermal cycle used was as follows: 95°C for 3 min followed by 45 cycles of 95°C for 30 s, 60°C for 30 s, and 72°C for 40 s. All gene-specific primers for q-RT PCR were designed on the basis of the cDNA sequences and listed in Supplementary Table S1. The specific primer for the rice actin gene (AK070531) was used as an internal control. The primers were designed using Primer Express Software (Foster City, CA, USA) and checked using the BLAST program with the rice genomic sequence available in the database of the Institute for Genomic Research (TIGR, http://rice.plantbiology.msu.edu/) to ensure that the primers would amplify a unique and desired cDNA segment. The specificity of the reactions was checked by melting curve analysis, and three replicates of each cDNA sample were used for qRT-PCR analysis.

## Results

### Natural light/dark cycle regulation of photosynthetic parameters

**Figure 1:**
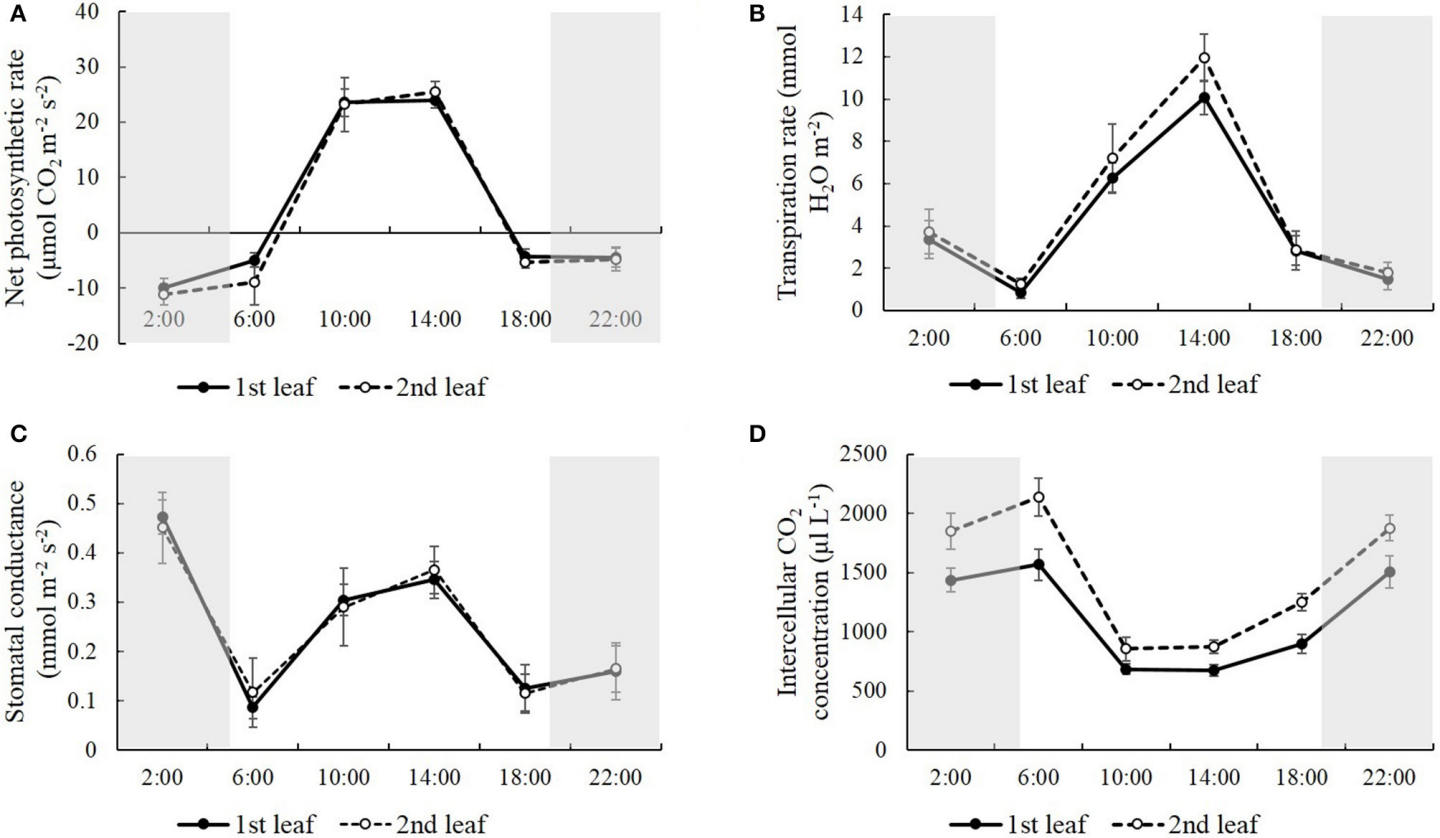
**The net photosynthetic rate (A), transpiration rate (B), stomatal conductance (C), and intercellular CO_2_ concentration (D) in the first and second leaves from the top of the rice plant at the time of 2:00, 6:00, 10:00, 14:00, 18:00, and 22:00**. Values are mean ± *SD* from 10 randomly selected plants.

**Figure 2:**
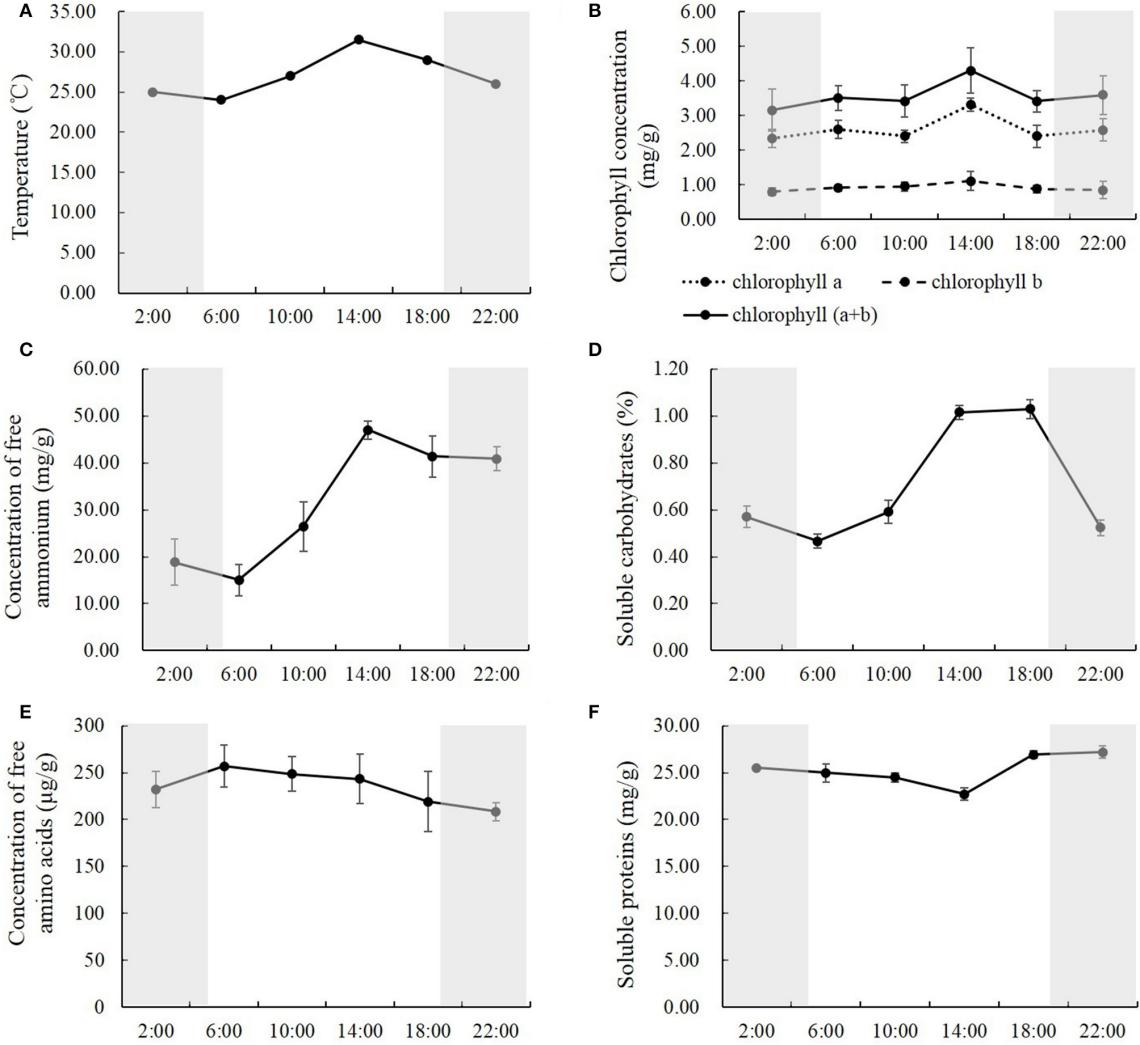
**The temperature (A), the concentrations of chlorophyll (B), free ammonium (C) and amino acids (E), soluble carbohydrates (D), and proteins (F) in the rice shoots at the time of 2:00, 6:00, 10:00, 14:00, 18:00, and 22:00**. Values are mean ± *SD* from three biological replicates.

In order to study how natural light/dark cycle affected rice photosynthesis, we tested the photosynthetic parameters, including net photosynthetic rate, transpiration rate, stomatal conductance, and intercellular CO_2_ concentration, in the first and second leaves from the top of the rice plant at the times: 2:00, 6:00, 10:00, 14:00, 18:00, and 22:00, respectively (Figure 1). We also monitored the temperature when we harvested the samples at each time point. The temperature was 25.0°C at 2:00, 24.0°C at 6:00, 27.0°C at 10:00, 31.5°C at 14:00, 29.0°C at 18:00 and 26.0°C at 22:00, and there was an obvious peak of high temperature, (31.5°C) at 14:00 in the daytime (Figure 2A). Generally, no significant differences were observed in the net photosynthetic rate, transpiration rate, and stomatal conductance, between the first and second leaves from the top of the rice plant, while the intercellular CO_2_ concentration in the second leaf was 24.6–39.3% higher than the one in the first leaf at each time point (Figure 1D). The net photosynthetic rate reached the highest point at 10:00 and kept stable from 10:00 to 14:00, while it fell below zero at the other four time points, which means that the respiratory rate was greater than the photosynthetic rate at these time points (Figure 1A). Figure 1A also indicated that the photosynthetic rate was highest at mid-day (from 10:00 to 14:00), while the respiratory rate was highest at mid-night (2:00). There was an obvious high peak of transpiration rate in the daytime, which reached the highest point at 14:00, while there was a slight change-like sine wave from 18:00 to 6:00 (Figure 1B). Similarly, a high peak of stomatal conductance was observed in the daytime; however, the highest stomatal conductance was shown after 2:00 (Figure 1C). Conversely, the intercellular CO_2_ concentration during the nighttime was significantly higher than in the daytime, which displayed a low valley in the daytime. This concentration was lowest at 10:00, and kept stable from 10:00 to 14:00, while it increased and kept relatively constant during the night until 6:00 (Figure 1D).

### Natural light/dark cycle regulation of carbon-nitrogen metabolites

To study how carbon-nitrogen metabolites were regulated by the natural light/dark cycle, we analyzed the concentrations of chlorophyll, free ammonium and amino acids, soluble proteins, and carbohydrates in the rice shoots. Similar results were observed within the concentrations of chlorophyll, free ammonium, and soluble carbohydrates. The concentrations of chlorophyll a, chlorophyll b, and chlorophyll (a+b) were relatively constant at the times of 2:00, 6:00, 10:00, 18:00, and 22:00, while there was a high peak at the time of 14:00 (Figure 2B). Compared to the other time points, at the time of 14:00, the concentration of chlorophyll a was 27.4–42.2% higher, the concentration of chlorophyll b was 16.8–38.8% higher, and the concentration of chlorophyll (a+b) was 19.8–36.3% higher (Figure 2B). For the concentration of free ammonium, there were no significant changes between the first two time points (2:00 and 6:00), and between the last two time points (18:00 and 22:00) at nighttime, while significant changes were observed from 6:00 to 18:00 in the daytime (Figure 2C). The free ammonium concentration increased by 76.5 and 77.4% from 6:00 to 10:00 and from 10:00 to 14:00, respectively; and it decreased by 12.0% from 14:00 to 18:00 (Figure 2C). For the soluble carbohydrates, there were no significant changes between 22:00 to 2:00 during the night, while significant changes were observed from 6:00 to 22:00 (Figure 2D). The soluble carbohydrates increased by 26.8 and 71.8% from 6:00 to 10:00 and from 10:00 to 14:00, respectively, and kept stable from 14:00 to 18:00, then declined by 49.2% from 18:00 to 22:00 (Figure 2D).

However, the concentration of free amino acids and soluble proteins showed slight changes among the different time points during the entire day. The concentrations of free amino acids were relatively constant from the time of 2:00 to 22:00 and were slightly higher in the daytime than at nighttime (Figure 2E). Compared to nighttime, the average concentration of free amino acids in the daytime was 9.9% higher, and the highest one at 6:00 was 23.3% higher than the lowest one at 22:00 (Figure 2E). Opposite results were observed in the soluble proteins; they were slightly lower in the daytime than at night (Figure 2F).The average concentration of soluble proteins in the daytime was 6.0% lower than at night, and the lowest one at 14:00, was 16.5% lower than the highest one at 22:00 (Figure 2F).

### Natural light/dark cycle regulation of nitrogen metabolic enzymes

**Figure 3:**
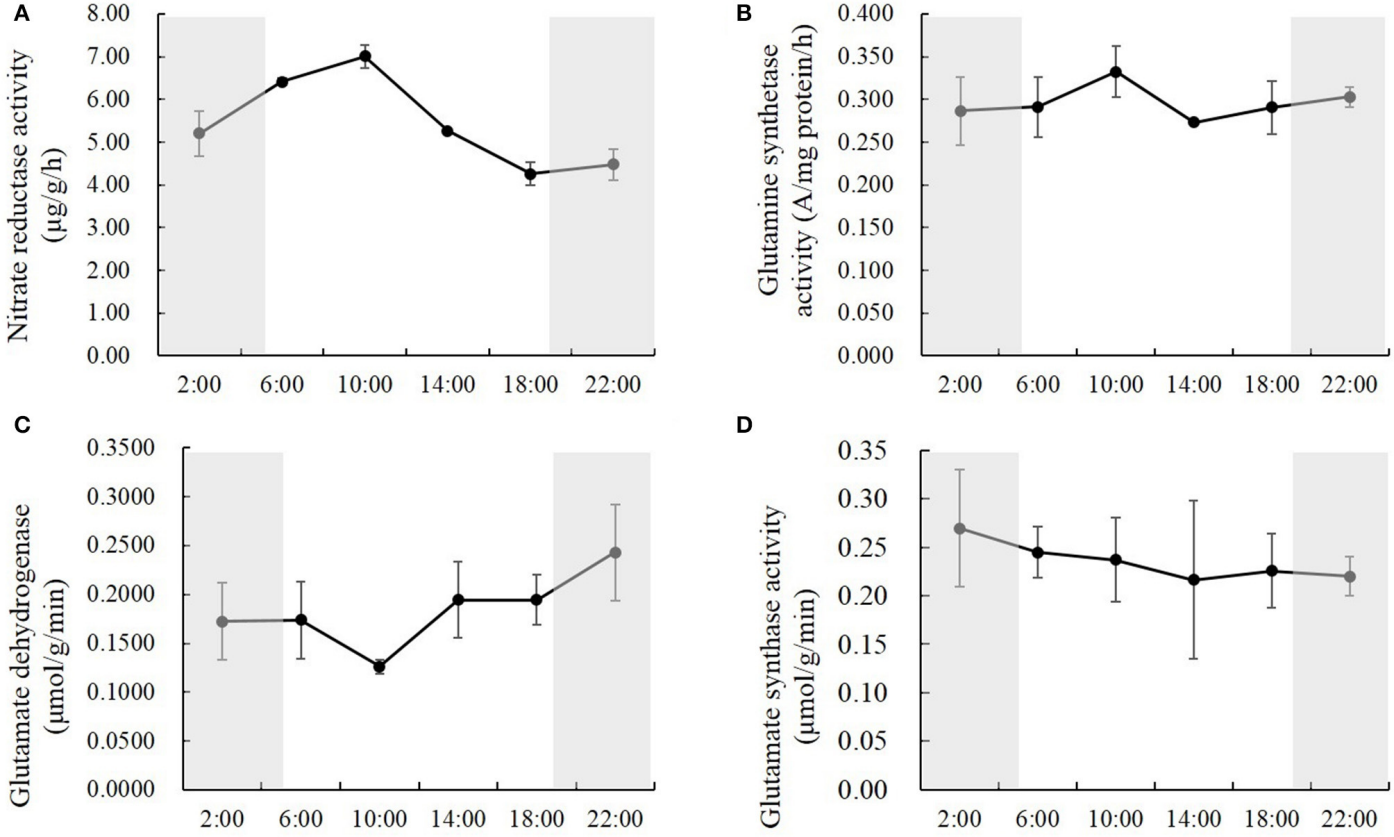
**The activities of nitrate reductase (A), glutamine synthetase (B), glutamate dehydrogenase (C), and glutamate synthase (D) in the rice shoots at the time of 2:00, 6:00, 10:00, 14:00, 18:00, and 22:00**. Values are mean ± *SD* from three biological replicates.

To study how nitrogen metabolic enzymes were regulated by the natural light/dark cycle, we analyzed the activities of nitrate reductase, glutamine synthetase, glutamate dehydrogenase and glutamate synthase in the rice shoots at the times of 2:00, 6:00, 10:00, 14:00, 18:00, and 22:00. Various, changing patterns were observed for the activities of nitrate reductase, glutamine synthetase, glutamate dehydrogenase, and glutamate synthase. For nitrate reductase, the activities changed significantly at the different time points, and the activities in the daytime were higher than those at night (Figure 3A). The nitrate reductase activity increased by 23.1 and 9.3% from 2:00 to 6:00 and from 6:00 to 10:00, respectively; and decreased by 24.8 and 19.1% from 10:00 to 14:00 and from 14:00 to 18:00, respectively; it kept relatively stable at the early part of the night (Figure 3A). There were no significant changes in glutamine synthetase activities at the different time points, except that it was the highest at 10:00, which was 21.7% higher than the lowest reading (Figure 3B). The opposite result displayed in the glutamate dehydrogenase activity. There were no significant changes of glutamate dehydrogenase activities at the different time points, except that it was the lowest at 10:00, which was 48.1% lower than the highest one (Figure 3C). However, the glutamate synthase activities kept constant at all of the different time points (Figure 3D).

### Natural light/dark cycle regulation of carbon-nitrogen metabolic genes

To study how gene expression responds to the natural light/dark cycle, the transcriptome was also analyzed in the rice shoots samples harvested at times: 2:00, 6:00, 10:00, 14:00, 18:00, and 22:00. A total of 397,173,322 high-quality reads (average length = 100 bp) were generated using an Illumina Hiseq 2000 sequencer (Supplementary Table S2). Each sample was represented by an average of 66.2 million reads, ~98.3% of the reads could be mapped to the rice genome reference, and of these mapped reads, ~95.0% mapped uniquely (Supplementary Table S2). Supplementary Figure S2 shows the reads mapped to individual chromosomes at each time point. Each sample was also represented by an average of 81.6 million mapped events, ~87.8% of the events could be mapped to genes, and of these mapped events, ~97.1% mapped to exons (Supplementary Table S3). Supplementary Figures S3, S4 display the heat map and functional categories of these mapped genes, respectively. The eggnog functional category analysis revealed that more than 1/3 (18.64 + 16.71%) of these genes were described to function unknown, or general function prediction only, more than 30% (9.21 + 7.87 + 7.74 + 6.03%) were described to DNA and protein regulation and signal transduction, 4.42% were described to carbohydrate transport and metabolism, and 3.53% were described to amino acid transport and metabolism (Supplementary Figure S4). Additionally, a total of 78,306 alternative splicing events have been found in our study, which could be divided into four groups: alternative 5′ donor sites (10.73%), alternative 3′ acceptor sites (19.88%), intron retention (64.92%), and exon skipping (4.47%) (Supplementary Figure S5). These alternative splicing events could be mapped to a total of 54,766 genes, 13.98% of these genes belong to the events of alternative 5′ donor sites, 24.27% belong to the events of alternative 3′ acceptor sites, 56.03% belong to the events of intron retention and 5.72% belong to the events of exon skipping (Supplementary Figure S5).

**Figure 4:**
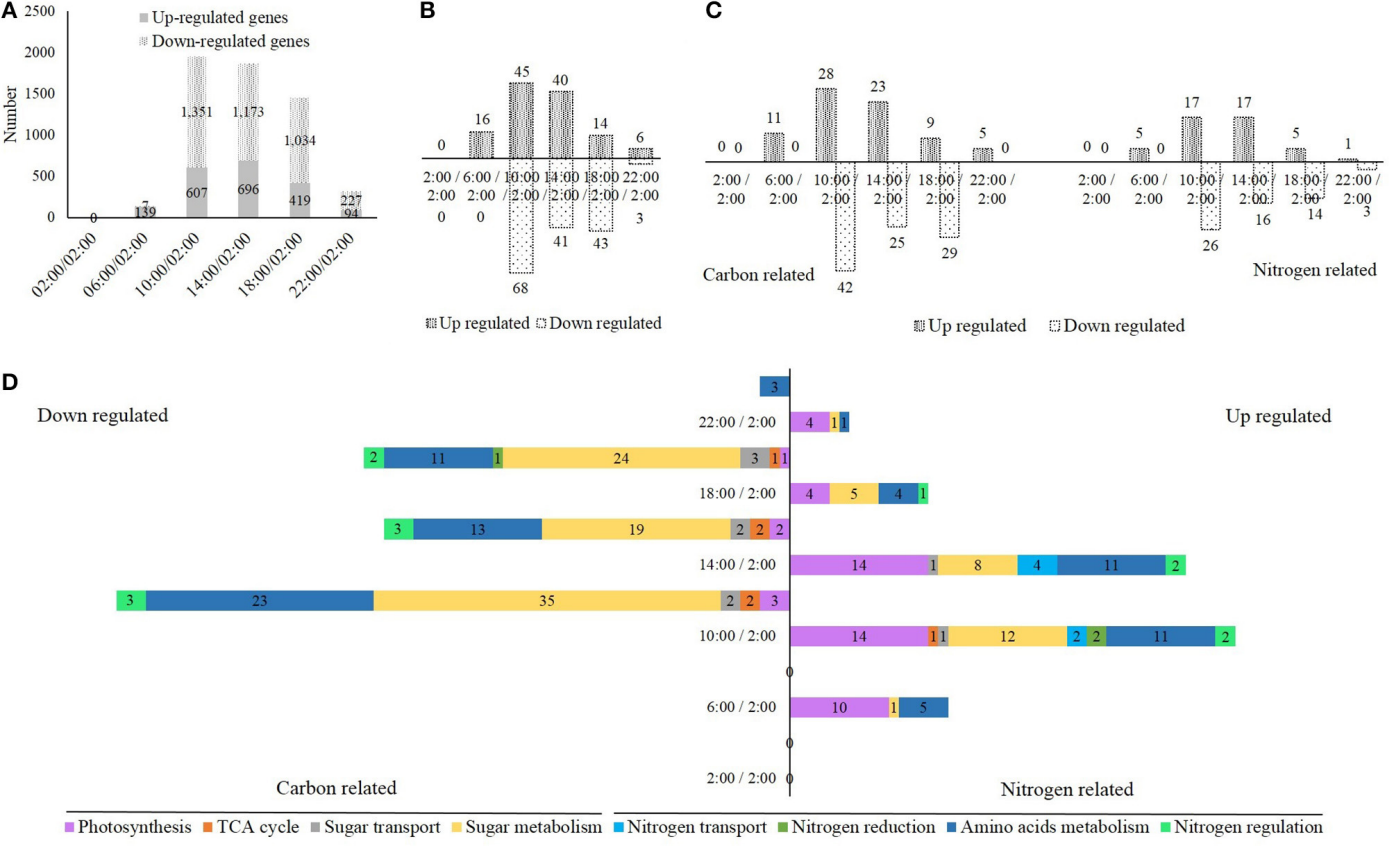
**(A)** The number of up- and down-regulated genes in the rice shoots at the time of 2:00, 6:00, 10:00, 14:00, 18:00, and 22:00 when compared to the time of 2:00. **(B,C)** The number of up- and down-regulated carbon and nitrogen metabolism related genes at the time of 2:00, 6:00, 10:00, 14:00, 18:00, and 22:00 when compared to the time of 2:00. **(D)** The number of up- and down-regulated genes, which involved in photosynthesis, TCA cycle, sugar transport, sugar metabolism, nitrogen transport, nitrogen reduction, amino acids metabolism, and nitrogen regulation, at the time of 2:00, 6:00, 10:00, 14:00, 18:00, and 22:00 when compared to the time of 2:00.

Compared to the mid-night time point (2:00), we obtained 146 (139 up- + 7 down-regulated), 1958 (607 up- + 1351 down-regulated), 1869 (696 up- + 1173 down-regulated), 1453 (419 up- + 1034 down-regulated), and 321 (94 up- + 227 down-regulated) differentially expressed genes at the times 6:00, 10:00, 14:00, 18:00, and 22:00, respectively (Figure 4A). Several randomly selected differentially expressed genes were also analyzed by qRT-PCR to validate the RNAseq results, and a close correlation was observed in this study (Supplementary Figure S6). Next, the carbon and nitrogen metabolism-related differentially expressed genes were selected for further analysis. We obtained 16 (16 up-regulated), 113 (45 up- + 68 down-regulated), 81 (40 up- + 41 down-regulated), 54 (14 up- + 43 down-regulated), and 9 (6 up- + 3 down-regulated) carbon and nitrogen metabolic differentially expressed genes at the times of 6:00, 10:00, 14:00, 18:00, and 22:00, respectively (Figure 4B). The number of these differentially expressed genes was highest at 10:00, and declined gradually from 14:00 to 22:00, which closely correlated to the light and temperature (Figures 4A,B). More down-regulated genes than up-regulated genes and more carbon-related genes than nitrogen-related genes were observed (Figures 4B,C). Additionally, we divided these carbon and nitrogen metabolic differentially expressed genes into eight groups: photosynthesis, TCA cycle, sugar transport, sugar metabolism, nitrogen transport, nitrogen reduction, amino acids metabolism, and nitrogen regulation (Figure 4D). For down-regulated genes, most were involved in the sugar and amino acid metabolism, while for up-regulated genes, most were involved in the photosynthesis, sugar, and amino acid metabolism (Figure 4D). We also listed these carbon and nitrogen metabolic differentially expressed genes at each time point in Tables 1–5.

**Table 1:** **Differentially expressed genes involved in carbon and nitrogen metabolism at the time of 6:00 compared to 2:00**.

	**ID number**	**Gene annotation**	**Base mean 6:00**	**Base mean 2:00**	**Fold change (6:00/2:00)**
**CARBON RELATED**					
Photosynthesis	OS04G0414700	PsaO; photosystem I subunit PsaO	20251.84	824.87	24.55
	OS06G0320500	LHCA1; light-harvesting complex I chlorophyll a/b binding protein 1	21176.03	1192.63	17.76
	OS02G0197600	LHCA3; light-harvesting complex I chlorophyll a/b binding protein 3	48958.24	1555.22	31.48
	OS08G0435900	LHCA4; light-harvesting complex I chlorophyll a/b binding protein 4	47573.59	1240.63	38.35
	OS01G0600900	LHCB1; light-harvesting complex II chlorophyll a/b binding protein 1	25126.92	897.98	27.98
	OS09G0346500	LHCB1; light-harvesting complex II chlorophyll a/b binding protein 1	17529.23	200.13	87.59
	OS03G0592500	LHCB2; light-harvesting complex II chlorophyll a/b binding protein 2	29101.71	466.71	62.36
	OS07G0562700	LHCB3; light-harvesting complex II chlorophyll a/b binding protein 3	15376.76	655.02	23.48
	OS11G0242800	LHCB5; light-harvesting complex II chlorophyll a/b binding protein 5	36926.38	1932.58	19.11
	OS04G0457000	LHCB6; light-harvesting complex II chlorophyll a/b binding protein 6	24346.09	1030.90	23.62
TCA cycle	–				
Sugar transport	–				
Sugar metabolism	OS05G0366600	Beta-glucosidase 22	8012.55	409.85	19.55
**NITROGEN RELATED**					
Nitrogen transport	–				
Nitrogen reduction	–				
Amino acids metabolism	OS09G0424200	Glutamine synthetase-like protein	198.74	4.43	44.85
	OS03G0231600	Branched-chain amino acid aminotransferase	3405.72	130.71	26.06
	OS12G0434400	CARP, pepA; leucyl aminopeptidase	40.18	0.74	54.41
	OS03G0283000	GST; glutathione S-transferase	130.32	1.48	88.24
	OS05G0148900	GST; glutathione S-transferase	180.28	5.17	34.88
Nitrogen regulation	–				

**Table 2:** **Differentially expressed genes involved in carbon and nitrogen metabolism at the time of 10:00 compared to 2:00**.

	**ID number**	**Gene annotation**	**Base mean 10:00**	**Base mean 2:00**	**Fold change (10:00/2:00)**
**CARBON RELATED**					
Photosynthesis	OS04G0414700	psaO; photosystem I subunit PsaO	69758.06	824.87	84.57
	OS02G0578400	psbQ; photosystem II oxygen-evolving enhancer protein 3	14.27	468.19	0.03
	OS07G0148900	psaK; photosystem I subunit X	75436.40	3641.39	20.72
	OS06G0320500	LHCA1; light-harvesting complex I chlorophyll a/b binding protein 1	87592.06	1192.63	73.44
	OS07G0577600	LHCA2; light-harvesting complex I chlorophyll a/b binding protein 2	98396.61	1905.99	51.62
	OS02G0197600	LHCA3; light-harvesting complex I chlorophyll a/b binding protein 3	154389.67	1555.22	99.27
	OS08G0435900	LHCA4; light-harvesting complex I chlorophyll a/b binding protein 4	182166.49	1240.63	146.83
	OS01G0600900	LHCB1; light-harvesting complex II chlorophyll a/b binding protein 1	77248.34	897.98	86.02
	OS09G0346500	LHCB1; light-harvesting complex II chlorophyll a/b binding protein 1	187861.96	200.13	938.72
	OS03G0592500	LHCB2; light-harvesting complex II chlorophyll a/b binding protein 2	136806.77	466.71	293.13
	OS07G0562700	LHCB3; light-harvesting complex II chlorophyll a/b binding protein 3	90175.85	655.02	137.67
	OS07G0558400	LHCB4; light-harvesting complex II chlorophyll a/b binding protein 4	153346.74	3334.19	45.99
	OS11G0242800	LHCB5; light-harvesting complex II chlorophyll a/b binding protein 5	152479.30	1932.58	78.90
	OS04G0457000	LHCB6; light-harvesting complex II chlorophyll a/b binding protein 6	52804.34	1030.90	51.22
	OS03G0129300	GAPA; glyceraldehyde-3-phosphate dehydrogenase	227473.42	13350.06	17.04
	OS02G0152400	rbcS; ribulose-bisphosphate carboxylase small chain	14.27	807.15	0.02
	OS01G0758300	ppc; phosphoenolpyruvate carboxylase	2.85	280.62	0.01
TCA cycle	OS05G0573200	IDH1, IDH2, icd; isocitrate dehydrogenase	17.12	584.13	0.03
	OS04G0551200	MDH1; malate dehydrogenase	2.85	356.68	0.01
	OS07G0630800	MDH2; malate dehydrogenase	2889.11	140.31	20.59
Sugar transport	OS03G0218400	Sugar carrier protein C	5517.13	290.22	19.01
	OS03G0363600	Sugar transporter family protein	1.43	90.09	0.02
	OS08G0535200	Bidirectional sugar transporter SWEET11	1.43	692.68	0.00
Sugar metabolism	OS07G0446800	Hexokinase-1	35.67	0.74	48.30
	OS11G0210600	adh; alcohol dehydrogenase	4.28	527.27	0.01
	OS02G0730000	Aldehyde dehydrogenase (NAD+)	18.55	520.62	0.04
	OS04G0540600	Aldehyde dehydrogenase (NAD+)	9.99	1436.32	0.01
	OS10G0155500	galM; aldose 1-epimerase	114.14	1934.05	0.06
	OS01G0658700	Glucose-6-phosphate 1-epimerase	32.81	1368.38	0.02
	OS08G0154300	talA, talB; transaldolase	95.59	1668.94	0.06
	OS01G0312500	Pectinesterase	11.41	644.68	0.02
	OS05G0521600	Pectinesterase	4.28	167.63	0.03
	OS07G0675100	Pectinesterase	2.85	203.82	0.01
	OS10G0457200	pel; pectate lyase	1.43	142.52	0.01
	OS12G0443500	UGDH, ugd; UDP-glucose 6-dehydrogenase 4	356.68	6183.21	0.06
	OS03G0268400	GMPP; mannose-1-phosphate guanylyltransferase	45.66	2013.07	0.02
	OS06G0652300	fcl; GDP-L-fucose synthase 2	216.86	5.17	41.95
	OS02G0714200	pfk; pyrophosphate-fructose-6-phosphate 1-phosphotransferase	235.41	4568.91	0.05
	OS11G0171300	ALDO; fructose-bisphosphate aldolase, class I	799472.65	45409.98	17.61
	OS04G0413500	sacA; beta-fructofuranosidase, insoluble isoenzyme 2	4.28	609.98	0.01
	OS08G0409100	Probable trehalose-phosphate phosphatase 6	5957.98	250.34	23.80
	OS07G0694700	L-ascorbate peroxidase 2, cytosolic	64402.15	2838.68	22.69
	OS02G0831500	Sucrose synthase 6	38.52	1574.42	0.02
	OS03G0401300	Sucrose synthase	1111.42	110538.58	0.01
	OS04G0309600	Sucrose synthase 5	9.99	310.90	0.03
	OS05G0365600	Beta-glucosidase 19	17330.37	849.98	20.39
	OS05G0366600	Beta-glucosidase 22	17453.07	409.85	42.58
	OS09G0491100	Beta-glucosidase 30	2.85	5504.55	0.00
	OS10G0323500	Beta-glucosidase 34	1.43	68.68	0.02
	OS04G0640700	XYL4; beta-D-xylosidase 4	7.13	3069.82	0.00
	OS07G0681700	GAUT; alpha-1,4-galacturonosyltransferase	28.53	1561.13	0.02
	OS05G0580000	glgC; glucose-1-phosphate adenylyltransferase	196.89	4688.54	0.04
	OS07G0243200	glgC; glucose-1-phosphate adenylyltransferase	11.41	245.17	0.05
	OS09G0298200	glgC; glucose-1-phosphate adenylyltransferase	71.34	1664.51	0.04
	OS07G0489200	Putative UDP-glucoseglucosyltransferase	154.09	2.95	52.16
	OS01G0969100	Putative dTDP-glucose 4,6-dehydratase	139.82	2758.92	0.05
	OS10G0565200	Beta-amylase	49.94	1520.51	0.03
	OS03G0736300	Endoglucanase 10	649.16	15384.54	0.04
	OS02G0605900	Chitinase 6	609.21	31.02	19.64
	OS04G0493400	Chitinase	1.43	1242.11	0.00
	OS11G0462100	Chitinase	1.43	538.34	0.00
	OS05G0415700	HEXA_B; hexosaminidase	21.40	2411.84	0.01
	OS04G0613700	UAP1; UDP-N-acetylglucosamine pyrophosphorylase	107.00	3476.72	0.03
	OS07G0181000	PK, pyk; pyruvate kinase	171.21	2948.71	0.06
	OS04G0623500	HAO; (S)-2-hydroxy-acid oxidase	82.75	2419.97	0.03
	OS03G0131200	katE, CAT, catB, srpA; catalase	153537.92	6428.38	23.88
	OS07G0658700	PIP5K; 1-phosphatidylinositol-4-phosphate 5-kinase	21.40	488.13	0.04
	OS05G0127200	PLCD; phosphatidylinositol phospholipase C, delta	54.22	1220.69	0.04
	OS04G0691900	PIKFYVE, FAB1; 1-phosphatidylinositol-3-phosphate 5-kinase	1366.80	31.02	44.07
	OS03G0726200	ITPK1; inositol-1,3,4-trisphosphate 5/6-kinase/inositol-tetrakisphosphate 1-kinase	3886.39	180.93	21.48
**NITROGEN RELATED**					
Nitrogen transport	OS06G0228500	Putative amino acid transport protein	206.87	10.34	20.01
	OS11G0298000	Transmembrane amino acid transporter protein	201.17	4.43	45.40
Nitrogen reduction	OS02G0770800	Nitrate reductase	4886.52	26.58	183.81
	OS01G0357100	nirA; ferredoxin-nitrite reductase	36312.88	2064.76	17.59
Amino acids metabolism	OS09G0424200	Glutamine synthetase-like protein	596.37	4.43	134.60
	OS03G0338000	AGXT2; alanine-glyoxylate transaminase/(R)-3-amino-2-methylpropionate-pyruvate transaminase	4.28	128.49	0.03
	OS09G0294000	thrA; bifunctional aspartokinase/homoserine dehydrogenase 1	54191.11	2123.84	25.52
	OS01G0711400	GLDC, gcvP; glycine dehydrogenase	141399.39	8744.22	16.17
	OS03G0196600	cysE; serine O-acetyltransferase	831.78	46.52	17.88
	OS01G0814800	cysK; cysteine synthase A	15.69	646.16	0.02
	OS10G0422200	mmuM; homocysteine S-methyltransferase	7.13	262.16	0.03
	OS09G0294000	thrA; bifunctional aspartokinase/homoserine dehydrogenase 1	54191.11	2123.84	25.52
	OS02G0237100	SRM, speE; spermidine synthase	4.28	167.63	0.03
	OS07G0182900	DNMT, dcm; DNA (cytosine-5-)-methyltransferase	17.12	755.45	0.02
	OS10G0104900	DNMT, dcm; DNA (cytosine-5-)-methyltransferase	15.69	1553.00	0.01
	OS09G0451400	1-aminocyclopropane-1-carboxylate oxidase 1	45.66	1350.66	0.03
	OS04G0107600	Arginine decarboxylase	2.85	108.56	0.03
	OS11G0644800	TAT; tyrosine aminotransferase	5250.33	125.54	41.82
	OS02G0168100	HPD, hppD; 4-hydroxyphenylpyruvate dioxygenase	6545.79	371.45	17.62
	OS01G0770200	Tyrosine decarboxylase	238.26	11.08	21.51
	OS01G0263300	Peroxidase	111.28	2220.58	0.05
	OS01G0543100	Peroxidase	11.41	457.85	0.02
	OS02G0833900	Peroxidase	14.27	677.18	0.02
	OS03G0234900	Class III peroxidase 39; Peroxidase 53	1.43	1168.26	0.00
	OS03G0762400	Class III peroxidase 51; Peroxidase 35	5.71	146.22	0.04
	OS04G0498700	Peroxidase	1.43	403.94	0.00
	OS04G0656800	Peroxidase	5.71	152.86	0.04
	OS06G0274800	Class III peroxidase 77; Peroxidase 49	5.71	412.80	0.01
	OS06G0681600	Peroxidase	17.12	367.02	0.05
	OS09G0507500	Class III peroxidase 123	4.28	175.02	0.02
	OS02G0626100	PTAL; phenylalanine/tyrosine ammonia-lyase	796.11	14801.89	0.05
	OS04G0614500	Probable mitochondrial gamma-aminobutyrate transaminase 2	1.43	142.52	0.01
	OS05G0148900	GST; glutathione S-transferase	226.85	5.17	43.88
	OS10G0528200	Glutathione S-transferase GSTU6	1436.71	24.37	58.96
	OS10G0530400	Glutathione S-transferase GSTU6	8294.95	395.82	20.96
	OS10G0528900	Glutathione S-transferase GSTU6	8.56	406.90	0.02
	OS06G0168600	RRM1; ribonucleoside-diphosphate reductase subunit M1	101.30	2138.61	0.05
	OS06G0127900	RRM2; ribonucleoside-diphosphate reductase subunit M2	8.56	258.46	0.03
Nitrogen regulation	OS02G0787600	Glutamate receptor	151.23	3.69	40.96
	OS03G0169600	Dof domain, zinc finger family protein	4731.00	240.74	19.65
	OS12G0569900	Dof domain, zinc finger family protein	4.28	118.16	0.04
	OS03G0371800	Myb-like DNA-binding domain containing protein	1.43	226.71	0.01
	OS05G0140100	MYB transcription factor; MYB16 protein	44.23	2244.21	0.02

**Table 3:** **Differentially expressed genes involved in carbon and nitrogen metabolism at the time of 14:00 compared to 2:00**.

	**ID number**	**Gene annotation**	**Base mean 14:00**	**Base mean 2:00**	**Fold change (14:00/2:00)**
**CARBON RELATED**					
Photosynthesis	OS04G0414700	psaO; photosystem I subunit PsaO	30894.18	824.87	37.45
	OS01G0860601	petF; ferredoxin	2738.23	144.74	18.92
	OS10G0389300	Putative red chlorophyll catabolite reductase	161.47	3.69	43.73
	OS06G0320500	LHCA1; light-harvesting complex I chlorophyll a/b binding protein 1	58262.97	1192.63	48.85
	OS07G0577600	LHCA2; light-harvesting complex I chlorophyll a/b binding protein 2	45059.13	1905.99	23.64
	OS02G0197600	LHCA3; light-harvesting complex I chlorophyll a/b binding protein 3	80870.39	1555.22	52.00
	OS08G0435900	LHCA4; light-harvesting complex I chlorophyll a/b binding protein 4	110823.63	1240.63	89.33
	OS01G0600900	LHCB1; light-harvesting complex II chlorophyll a/b binding protein 1	56452.22	897.98	62.87
	OS09G0346500	LHCB1; light-harvesting complex II chlorophyll a/b binding protein 1	103192.35	200.13	515.64
	OS03G0592500	LHCB2; light-harvesting complex II chlorophyll a/b binding protein 2	74013.77	466.71	158.59
	OS07G0562700	LHCB3; light-harvesting complex II chlorophyll a/b binding protein 3	40180.50	655.02	61.34
	OS07G0558400	LHCB4; light-harvesting complex II chlorophyll a/b binding protein 4	78095.64	3334.19	23.42
	OS11G0242800	LHCB5; light-harvesting complex II chlorophyll a/b binding protein 5	75359.34	1932.58	38.99
	OS04G0457000	LHCB6; light-harvesting complex II chlorophyll a/b binding protein 6	23949.14	1030.90	23.23
	OS02G0152400	rbcS; ribulose-bisphosphate carboxylase small chain	34.60	807.15	0.04
	OS01G0758300	ppc; phosphoenolpyruvate carboxylase	5.77	280.62	0.02
TCA cycle	OS05G0573200	IDH1, IDH2, icd; isocitrate dehydrogenase	2.88	584.13	0.00
	OS04G0551200	MDH1; malate dehydrogenase	7.69	356.68	0.02
Sugar transport	OS03G0218400	Sugar carrier protein C	10840.45	290.22	37.35
	OS05G0214300	Bidirectional sugar transporter SWEET3a	6.73	164.68	0.04
	OS08G0535200	Bidirectional sugar transporter SWEET11	1.92	692.68	0.00
Sugar metabolism	OS11G0210600	adh; alcohol dehydrogenase	19.22	527.27	0.04
	OS02G0730000	Aldehyde dehydrogenase (NAD+)	32.68	520.62	0.06
	OS04G0540600	Aldehyde dehydrogenase (NAD+)	27.87	1436.32	0.02
	OS04G0458600	galM; aldose 1-epimerase	60.55	1.48	41.00
	OS01G0658700	Glucose-6-phosphate 1-epimerase	18.26	1368.38	0.01
	OS04G0137100	pel; pectate lyase	11.53	704.50	0.02
	OS04G0413500	sacA; beta-fructofuranosidase, insoluble isoenzyme 2	1.92	609.98	0.00
	OS07G0694700	L-ascorbate peroxidase 2, cytosolic	44885.17	2838.68	15.81
	OS03G0401300	Sucrose synthase	1161.99	110538.58	0.01
	OS04G0309600	Sucrose synthase 5	11.53	310.90	0.04
	OS02G0831500	Sucrose synthase 6	39.41	1574.42	0.03
	OS04G0249500	Sucrose synthase 7	33.64	581.18	0.06
	OS05G0366600	Beta-glucosidase 22	8376.14	409.85	20.44
	OS09G0491100	Beta-glucosidase 30	1.92	5504.55	0.00
	OS04G0640700	XYL4; beta-D-xylosidase 4	147.05	3069.82	0.05
	OS07G0681700	GAUT; alpha-1,4-galacturonosyltransferase	88.42	1561.13	0.06
	OS07G0243200	glgC; glucose-1-phosphate adenylyltransferase	1.92	245.17	0.01
	OS01G0176000	Putative UDP-glucose flavonoid 7-O-glucosyltransferase	865.97	19.94	43.43
	OS07G0489200	Putative UDP-glucoseglucosyltransferase	82.66	2.95	27.98
	OS10G0565200	Beta-amylase	40.37	1520.51	0.03
	OS09G0530200	Endoglucanase 23	1.92	206.03	0.01
	OS01G0660200	Acidic class III chitinase OsChib3a	12940.49	84.92	152.38
	OS01G0687400	Chitinase	795.81	28.06	28.36
	OS02G0605900	Chitinase 6	491.13	31.02	15.83
	OS04G0493400	Chitinase	14.42	1242.11	0.01
	OS05G0399400	Chitinase	2.88	923.09	0.00
	OS05G0415700	HEXA_B; hexosaminidase	4.81	2411.84	0.00
**NITROGEN RELATED**					
Nitrogen transport	OS03G0838400	Ammonium transporter 3 member 2	2370.12	36.92	64.19
	OS02G0550800	Ammonium transporter 3 member 3	1521.45	49.48	30.75
	OS01G0209800	Putative amino acid permease	622.80	28.80	21.62
	OS08G0127100	Putative histidine amino acidtransporter	14278.37	321.23	44.45
Nitrogen reduction	–				
Amino acids metabolism	OS09G0424200	Glutamine synthetase-like protein	157.62	4.43	35.57
	OS03G0236200	gadB, gadA, GAD; glutamate decarboxylase	629.53	19.20	32.79
	OS10G0422200	mmuM; homocysteine S-methyltransferase	9.61	262.16	0.04
	OS02G0237100	SRM, speE; spermidine synthase	0.96	167.63	0.01
	OS10G0104900	DNMT, dcm; DNA (cytosine-5-)-methyltransferase	11.53	1553.00	0.01
	OS03G0106400	ilvE; branched-chain amino acid aminotransferase	7.69	181.66	0.04
	OS07G0638400	PRDX6; peroxiredoxin 6, 1-Cys peroxiredoxin	127.83	1.48	86.55
	OS01G0263300	Peroxidase	30.76	2220.58	0.01
	OS01G0543100	Peroxidase	13.46	457.85	0.03
	OS02G0833900	Peroxidase	7.69	677.18	0.01
	OS03G0234900	Class III peroxidase 39; Peroxidase 53	69.20	1168.26	0.06
	OS04G0656800	Peroxidase	4.81	152.86	0.03
	OS05G0499300	Peroxidase 1	1.92	585.61	0.00
	OS06G0695500	Class III peroxidase 90	31.72	943.03	0.03
	OS09G0507500	Class III peroxidase 123	1.92	175.02	0.01
	OS02G0626600	Phenylalanine ammonia-lyase	100.92	3.69	27.33
	OS08G0448000	4-coumarate-CoA ligase	448.84	22.15	20.26
	OS12G0434400	CARP, pepA; leucyl aminopeptidase	42.29	0.74	57.27
	OS01G0372400	GST, gst; glutathione S-transferase	187.42	5.91	31.72
	OS03G0283000	GST, gst; glutathione S-transferase	363.30	1.48	245.98
	OS09G0467200	GST, gst; glutathione S-transferase	9561.20	626.96	15.25
	OS10G0527800	GST, gst; glutathione S-transferase	1372.48	47.26	29.04
	OS10G0528100	GST, gst; glutathione S-transferase	577.63	7.38	78.22
	OS06G0127900	RRM2; ribonucleoside-diphosphate reductase subunit M2	13.46	258.46	0.05
Nitrogen regulation	OS02G0787600	Glutamate receptor	535.34	3.69	144.99
	OS03G0169600	Dof domain, zinc finger family protein	3593.62	240.74	14.93
	OS12G0569900	Dof domain, zinc finger family protein	4.81	118.16	0.04
	OS01G0229000	MYB23 protein	18.26	618.84	0.03
	OS05G0140100	MYB transcription factor; MYB16 protein	88.42	2244.21	0.04

**Table 4:** **Differentially expressed genes involved in carbon and nitrogen metabolism at the time of 18:00 compared to 2:00**.

	**ID number**	**Gene annotation**	**Base mean 18:00**	**Base mean 2:00**	**Fold change (18:00/2:00)**
**CARBON RELATED**					
Photosynthesis	OS06G0320500	LHCA1; light-harvesting complex I chlorophyll a/b binding protein 1	29258.26	1192.63	24.53
	OS08G0435900	LHCA4; light-harvesting complex I chlorophyll a/b binding protein 4	52503.16	1240.63	42.32
	OS09G0346500	LHCB1; light-harvesting complex II chlorophyll a/b binding protein 1	13554.67	200.13	67.73
	OS03G0592500	LHCB2; light-harvesting complex II chlorophyll a/b binding protein 2	9331.47	466.71	19.99
	OS02G0152400	rbcS; ribulose-bisphosphate carboxylase small chain	27.32	807.15	0.03
TCA cycle	OS05G0573200	IDH1, IDH2, icd; isocitrate dehydrogenase	3.64	584.13	0.01
Sugar transport	OS01G0700100	Bidirectional sugar transporter; SWEET2b	13.66	3137.02	0.00
	OS05G0214300	Bidirectional sugar transporter; SWEET3a	6.37	164.68	0.04
	OS08G0535200	Bidirectional sugar transporter; SWEET11	3.64	692.68	0.01
Sugar metabolism	OS06G0136600	ENO, eno; enolase	29311.99	1561.13	18.78
	OS11G0210600	adh; alcohol dehydrogenase	25.50	527.27	0.05
	OS04G0540600	Aldehyde dehydrogenase (NAD+)	3.64	1436.32	0.00
	OS01G0658700	Glucose-6-phosphate 1-epimerase	34.60	1368.38	0.03
	OS01G0312500	Pectinesterase	30.05	644.68	0.05
	OS01G0743200	Pectinesterase	3.64	91.57	0.04
	OS05G0521600	Pectinesterase	7.28	167.63	0.04
	OS07G0675100	Pectinesterase	3.64	203.82	0.02
	OS04G0137100	pel; pectate lyase	17.30	704.50	0.02
	OS12G0443500	UGDH, ugd; UDP-glucose 6-dehydrogenase 4	259.51	6183.21	0.04
	OS01G0969100	Putative dTDP-glucose 4,6-dehydratase	161.17	2758.92	0.06
	OS08G0545200	gutB; L-iditol 2-dehydrogenase	9593.71	624.01	15.37
	OS08G0374800	galE, GALE; UDP-glucose 4-epimerase	320.52	4829.59	0.07
	OS02G0106100	sacA; beta-fructofuranosidase	7.28	1005.80	0.01
	OS02G0831500	Sucrose synthase 6	96.52	1574.42	0.06
	OS03G0401300	Sucrose synthase	4201.35	110538.58	0.04
	OS09G0491100	Beta-glucosidase 30	8.20	5504.55	0.00
	OS05G0580000	glgC; glucose-1-phosphate adenylyltransferase	270.44	4688.54	0.06
	OS07G0243200	glgC; glucose-1-phosphate adenylyltransferase	1.82	245.17	0.01
	OS01G0660200	Acidic class III chitinase; OsChib3a	1282.08	84.92	15.10
	OS04G0493400	Chitinase	14.57	1242.11	0.01
	OS04G0494100	Chitinase	40.06	979.95	0.04
	OS05G0399400	Chitinase	16.39	923.09	0.02
	OS11G0462100	Chitinase; Glycosyl hydrolases family 18 protein	2.73	538.34	0.01
	OS01G0891000	HEXA_B; beta-hexosaminidase	0.91	53.91	0.02
	OS05G0415700	HEXA_B; beta-hexosaminidase	18.21	2411.84	0.01
	OS02G0115700	CAT, catB, srpA; catalase isozyme A	27821.39	1810.73	15.36
	OS09G0521400	Putative hydroxymethylglutaryl coenzyme A synthase	163.90	3135.54	0.05
	OS03G0726200	ITPK1; inositol-1,3,4-trisphosphate 5/6-kinase/inositol-tetrakisphosphate 1-kinase	6105.34	180.93	33.75
**NITROGEN RELATED**					
Nitrogen transport	–				
Nitrogen reduction	OS08G0468100	Nitrate reductase	29.14	579.70	0.05
Amino acids metabolism	OS05G0555600	Glutamate synthase 2 [NADH], chloroplastic	3342.68	27.32	122.34
	OS03G0798300	DNMT, dcm; DNA (cytosine-5-)-methyltransferase	0.91	66.46	0.01
	OS10G0104900	DNMT, dcm; DNA (cytosine-5-)-methyltransferase	5.46	1553.00	0.00
	OS03G0106400	ilvE; branched-chain amino acid aminotransferase	7.28	181.66	0.04
	OS02G0306401	Nicotianamine aminotransferase	898.73	48.00	18.72
	OS01G0543100	Peroxidase	8.20	457.85	0.02
	OS03G0234900	Class III peroxidase 39; Peroxidase 53	40.98	1168.26	0.04
	OS04G0498700	Peroxidase	0.91	403.94	0.00
	OS04G0656800	Peroxidase	0.91	152.86	0.01
	OS05G0499300	Peroxidase 1	13.66	585.61	0.02
	OS10G0536700	Class III peroxidase 128	11.84	252.56	0.05
	OS11G0661600	Peroxidase	0.91	278.40	0.00
	OS03G0283000	GST; glutathione S-transferase	48.26	1.48	32.68
	OS10G0528100	GST; glutathione S-transferase	531.77	7.38	72.01
	OS06G0127900	RRM2; ribonucleoside-diphosphate reductase subunit M2	6.37	258.46	0.02
Nitrogen regulation	OS02G0787600	Glutamate receptor	124.75	3.69	33.79
	OS12G0569900	Dof domain, zinc finger family protein	0.91	118.16	0.01
	OS01G0229000	MYB23 protein	21.85	618.84	0.04

**Table 5:** **Differentially expressed genes involved in carbon and nitrogen metabolism at the time of 22:00 compared to 2:00**.

	**ID number**	**Gene annotation**	**Base mean 22:00**	**Base mean 2:00**	**Fold change (22:00/2:00)**
**CARBON RELATED**					
Photosynthesis	OS12G0207500	ATPF1B, atpD; F-type H+-transporting ATPase subunit beta	89.68	2.22	40.48
	OS05G0427800	rbcL; ribulose-bisphosphate carboxylase large chain	64.77	1.48	43.85
	OS10G0356000	rbcL; rRibulose bisphosphate carboxylase large chain	5478.44	110.03	49.79
	OS12G0207600	rbcL; ribulose-bisphosphate carboxylase large chain	1316.30	20.68	63.66
TCA cycle	–				
Sugar transport	–				
Sugar metabolism	OS07G0446800	Hexokinase-1	205.27	0.74	277.96
**NITROGEN RELATED**					
Nitrogen transport	–				
Nitrogen reduction	–				
Amino acids metabolism	OS07G0182900	DNMT, dcm; DNA (cytosine-5-)-methyltransferase	29.89	755.45	0.04
	OS10G0104900	DNMT, dcm; DNA (cytosine-5-)-methyltransferase	19.93	1553.00	0.01
	OS07G0677300	Peroxidase 2	1.00	281.36	0.00
	OS02G0626600	Phenylalanine ammonia-lyase	324.84	3.69	87.98
Nitrogen regulation	–				

### Natural light/dark cycle regulation of carbon-nitrogen related miRNAs and their target genes

**Figure 5:**
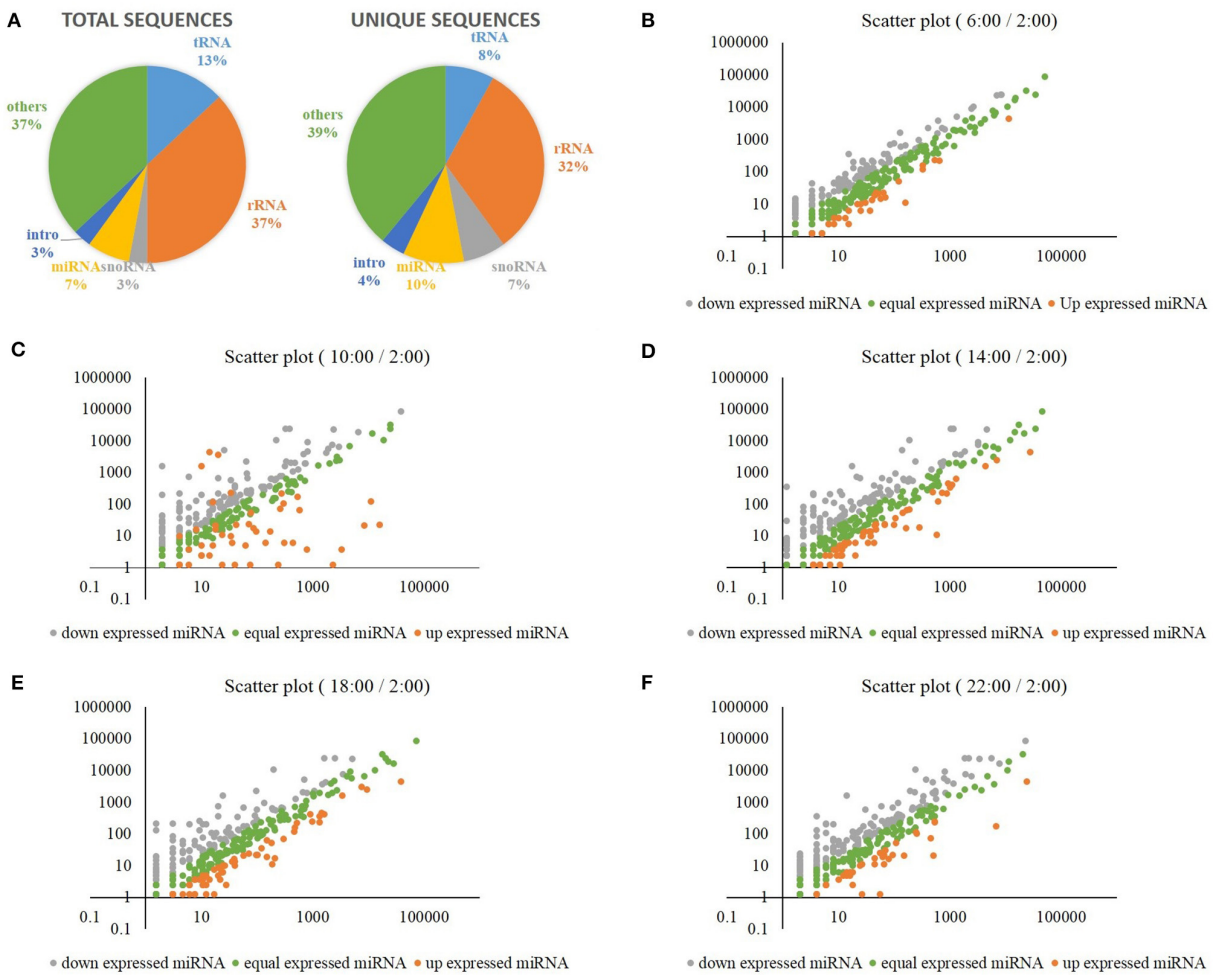
**(A)** The annotations of total and unique sRNA sequences, which were blasted with non-coding RNAs in the database of Rfam (11.0). **(B)** Differentially expressed mature miRNAs at the time of 6:00 when compared to the time of 2:00. **(C)** Differentially expressed mature miRNAs at the time of 10:00 when compared to the time of 2:00. **(D)** Differentially expressed mature miRNAs at the time of 14:00 when compared to the time of 2:00. **(E)** Differentially expressed mature miRNAs at the time of 18:00 when compared to the time of 2:00. **(F)** Differentially expressed mature miRNAs at the time of 22:00 when compared to the time of 2:00.

Additionally, the miRNAs were analyzed in the samples of rice shoots harvested at the times of 2:00, 6:00, 10:00, 14:00, 18:00, and 22:00. A total of 38,704,070 high-quality clean reads (15–30 nt) were generated using an Illumina Hiseq 2000 sequencer, and each sample was represented by an average of 6.5 million reads (Supplementary Table S4). Analysis of the size distribution of unique sequences showed that 24 nt long RNAs were the most abundant, followed by 23, 21, 22, and 25 nt long RNAs (Supplementary Figure S7). Figure 5A displays the annotations of total and unique sequences, which were blasted with non-coding RNAs in the database of Rfam (11.0). For unique sequences, more than 1/3 sRNA sequences matched rRNAs, followed by miRNAs (10%), tRNAs (8%), and snoRNAs (7%) (Figure 5A). By blasting with rice references in miRBase, we have detected a total of 2038 mature miRNAs, and each sample was represented by an average of 340 mature miRNAs (Supplementary Table S5). Supplementary Figure S8 displays the heat map of these mature miRNAs at each time point. When compared to the mid-night time point (2:00), we obtained differentially expressed mature miRNAs at the times of 6:00, 10:00, 14:00, 18:00, and 22:00, respectively (Figures 5B–F). Compared to the other time points, the variation of the mature miRNAs expression levels was greatest at the time of 10:00 (Figure 5C).

**Figure 6:**
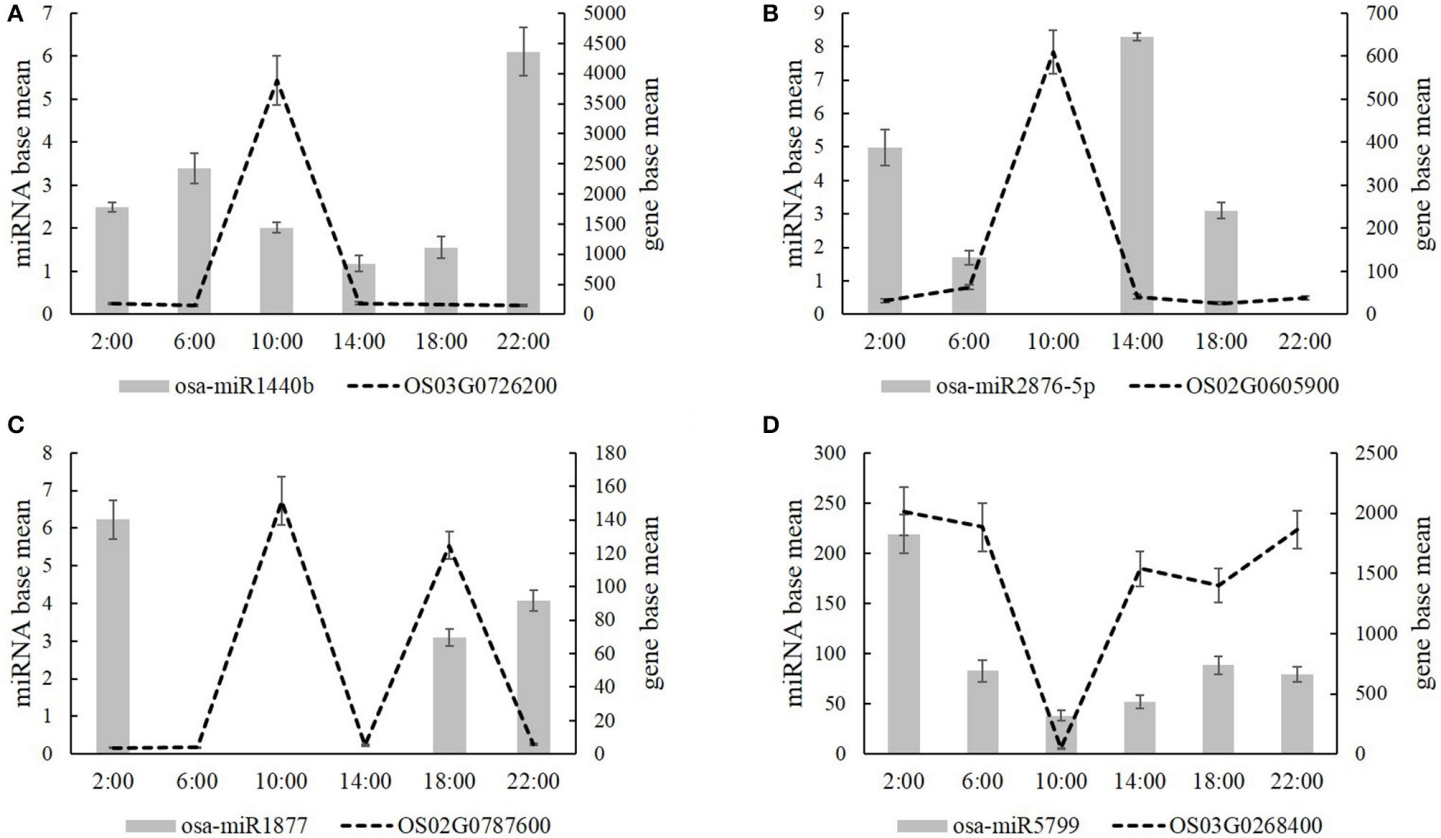
**The expression level of four carbon and nitrogen metabolism related miRNAs (A, Osa-miR1440b; B, osa-miR2876-5p; C, osa-miR1877; D, osa-miR5799) and their target genes at the time of 2:00, 6:00, 10:00, 14:00, 18:00, and 22:00**. Values are mean ± *SD* from three biological replicates.

Additionally, we analyzed the target genes of these mature miRNAs by psRNATarget. Next, we selected four carbon and nitrogen metabolism related miRNAs: osa-miR1440b, osa-miR2876-5p, osa-miR1877, and osa-miR5799, as they targeted the carbon and nitrogen metabolic differentially expressed genes, which are listed in Tables 1–5. Osa-miR1440b targeted the gene of inositol-1,3,4-trisphosphate 5/6-kinase/inositol-tetrakisphosphate 1-kinase (OS03G0726200), osa-miR2876-5p targeted the gene of chitinase 6 (OS02G0605900), osa-miR1877 targeted the gene of glutamate receptor (OS02G0787600), and osa-miR5799 targeted the gene of mannose-1-phosphate guanylyltransferase (OS03G0268400). Figure 6 shows the expression levels of osa-miR1440b, osa-miR2876-5p, osa-miR1877, osa-miR5799, and their target genes at each time points. The expression level analysis showed that the four carbon and nitrogen metabolis-related miRNAs negatively regulated their target genes at most of the time points (Figure 6). The same expression patterns of osa-miR1440b and osa-miR2876-5p target genes were observed; i.e., that they highly expressed at 10:00 and lowly expressed at other time points (Figures 6A,B). Conversely, the target gene of osa-miR5799 highly expressed at 2:00 and 22:00, expressed in the middle level at 6:00, 14:00, and 18:00, and expressed at the bottom level at 10:00 (Figure 6D). Additionally, the target gene of osa-miR1877 expressed highly at both 10:00 and 18:00, and expressed lowest at the other time points (Figure 6C). From the psRNATarget database, we found that these four carbon and nitrogen metabolism- related miRNAs also targeted several other genes (Supplementary Table S6).

## Discussion

For a notably long time, studies have focused on the analysis of circadian clock transgenic lines and mutants under artificially controlled light/dark conditions. The results clearly implied that the plant circadian clock influenced photosynthesis, growth and fitness as well as the primary metabolism, especially carbon metabolism (Green et al., 2002; Dodd et al., 2005; Yerushalmi et al., 2011). Generally, plants grow under the natural diurnal condition, where the temperature and light are dramatically changing and interacting with each other. However, little is known about the natural light/dark cycle regulation of plant metabolism and gene regulation. As cellular carbon and nitrogen metabolism must be tightly coordinated, in this paper, we focused on the natural light/dark cycle regulation of carbon and nitrogen metabolism, carbon and nitrogen related gene expression and miRNAs in rice.

Plants are photoautotrophs, which produce sugars from photosynthesis to ensure plant growth and development. It has been reported that photosynthesis is under circadian regulation in *Arabidopsis* and *Brassica rapa*. Compared to 24 h period cycles, more CO_2_ was fixed in *Arabidopsis* leaves grown under 28 h period cycles (Graf et al., 2010; Graf and Smith, 2011). Edwards et al. (2011) reported that photosynthesis rate, transpiration rate and stomatal conductance were significantly correlated with circadian period in *Brassica rapa*. In our study, the net photosynthetic rate, respiratory rate, stomatal conductance, and intercellular CO_2_ concentration were also regulated by the natural light/dark cycle. Similar regulatory patterns were shown in the net photosynthetic rate, respiratory rate, and stomatal conductance, such that an obvious high peak was observed in the daytime. However, the opposite regulatory pattern was shown in the intercellular CO_2_ concentration, which indicated that more CO_2_ was fixed into carbohydrates by a high photosynthetic rate in the daytime.

Metabolism is a crucial circadian output, which can ensure plants' optimal physiology, growth and behavior under the natural diurnal condition (Dodd et al., 2005; Turek et al., 2005). Metabolomics analysis has detected that ~30% of measured primary metabolites were under the regulation of circadian oscillations (Espinoza et al., 2010). Starch synthesis and degradation are important aspects of carbon metabolism in plants that are regulated by the circadian clock. Graf and Smith (2011) reported that starch degraded into sucrose, Suc6P (sucrose 6-phosphate), and Tre6P (trehalose 6-phosphate) at night in *Arabidopsis*, and sucrose arrived at a peak level in the morning, which was necessary to prevent sugar shortage and growth penalties at night. Significant correlations were also found in the day and nighttime between growth rates and the levels of Tre6P and shikimate, which is an intermediate in amino acid biosynthesis that may act as a read-out for amino acid biosynthesis (Seaton et al., 2014). Tre6P has been reported to inhibit SnRK1 (SNF1-related protein kinases 1), which provides a pathway to promote protein synthesis when sugars are high (Zhang et al., 2009; Paul et al., 2010; Nunes et al., 2013). In our study, there was a synchronic association between the temperature/light and the concentrations of chlorophyll a and soluble carbohydrates. However, the concentration of free amino acids and soluble proteins showed slight changes among the different time points during the entire day. These results suggest that higher temperature and light intensity in the mid-day promote carbon fixation, not protein synthesis. Additionally, a dramatic increase of free ammonium concentration and nitrate reductase activity were observed in the daytime, while the activities of glutamine synthetase, glutamate dehydrogenase, and glutamate synthase were slightly changed during the whole day. These results also suggest that the protein synthesis is not significantly facilitated by the light and temperature, although high levels of the free ammonium and carbohydrates were exhibited in the day time.

It has been reported that at least 30% of the genome transcripts are under circadian regulation (Harmer et al., 2000; Covington et al., 2008; Michael et al., 2008; Chow and Kay, 2013). Transcriptome analysis in *Arabidopsis* demonstrated the circadian regulation of key transcripts involved in photosynthesis and starch metabolism, isoprenoid (chlorophyll and carotene) biosynthesis, phenylpropanoid (flavonoid and anthocyanin) biosynthesis, redox balance and the membrane transport associated with nitrogen, sulfur and sugar (Dodd et al., 2005; Lu et al., 2005; Gutiérrez et al., 2008; Graf et al., 2010; Kerwin et al., 2011; Lai et al., 2012). In our study, the transcriptome was also analyzed in the samples of rice shoots harvested at the times of 2:00, 6:00, 10:00, 14:00, 18:00, and 22:00, to study how gene expression responds to the natural light/dark cycle. When compared to the middle-of-night time point (2:00), we obtained a large amount of differentially expressed genes at the times of 6:00, 10:00, 14:00, 18:00, and 22:00. Later, we focused on the analysis of carbon and nitrogen metabolism- related differentially expressed genes, which were divided into eight groups: photosynthesis, TCA cycle, sugar transport, sugar metabolism, nitrogen transport, nitrogen reduction, amino acid metabolism, and nitrogen regulation. Most of the down-regulated genes were involved in the sugar and amino acid metabolism, while most of the up-regulated genes were involved in the photosynthesis, sugar, and amino acid metabolism.

Post-transcriptional regulation can result in rapid and durable responses to the environmental light and temperature. The first evidence showing the importance of post-transcriptional regulation in maintaining circadian rhythmicity was discovered in the single-celled green algae, *Acetabularia*, which could survive for several weeks after removing its nucleus, and continue to photosynthesize rhythmically in constant light (Mergenhagen and Schweiger, 1975; Lakin-Thomas, 2006). Alternative pre-mRNA splicing and miRNA regulation are two processes that mediate most post-transcriptional regulation in response to environmental changes (Bartok et al., 2013). Recent studies demonstrated the importance of alternative splicing in mediating responses of circadian clock to temperature changes in *Arabidopsis* using a genome-wide approach (Bieniawska et al., 2008; Staiger and Green, 2011; James et al., 2012). The results revealed the extensive changes in the splicing of many clock genes. For example, a temperature-sensitive alternative splicing event involving intron retention in CCA1 mRNA was identified in *Arabidopsis* (Gould et al., 2006; Filichkin et al., 2010). It has also been reported that this alternative splicing regulation was present not only in *Arabidopsis thaliana* but also in *Oryza sativa, Brachypodiumdistachyon* and *Populustrichocarpa*, mono-, and di-cotyledonous species that diverged from a common ancestor 120–170 million years ago (Lynch and Conery, 2000; Tuskan et al., 2006). In our study, a total of 78,306 alternative splicing events have been found at each time point, which mainly belong to alternative 5′ donor sites, alternative 3′ acceptor sites, intron retention, and exon skipping. Additionally, there has been a growing awareness of the vital role played by miRNAs in regulating various aspects of circadian clock function. In our study, the miRNAs were analyzed in the samples of rice shoots harvested at the times of 2:00, 6:00, 10:00, 14:00, 18:00, and 22:00 through small RNA sequencing. When compared to the mid-night time point (2:00), we obtained differentially expressed mature miRNAs at the times of 6:00, 10:00, 14:00, 18:00, and 22:00. Four carbon and nitrogen metabolism-related miRNAs (osa-miR1440b, osa-miR2876-5p, osa-miR1877, and osa-miR5799) were found to be regulated by natural light/dark cycle. The expression level analysis showed that the four carbon and nitrogen metabolism-related miRNAs negatively regulated their target genes at most of the time points. These results may provide a good strategy to study how natural light/dark cycle regulates carbon and nitrogen metabolism to ensure plant growth and development.

## Author contributions

HC and FX designed the experiments, HL and ZL performed the experiments, HC and LS analyzed the data, GD contributed reagents, HC wrote the manuscript. All authors read and approved the manuscript.

## Funding

This work was supported in part by grants from the National Key Research and Development Program of China (2016YFD0200108), the Fundamental Research Funds for the Central Universities (2662013PY041).

### Conflict of interest statement

The authors declare that the research was conducted in the absence of any commercial or financial relationships that could be construed as a potential conflict of interest.

## Supplementary material

Supplementary Figure S1 **The diagram of rice germination, planting, and harvesting**.

Supplementary Figure S2 **The reads mapped to individual chromosome at the time of 2:00, 6:00, 10:00, 14:00, 18:00, and 22:00**.

Supplementary Figure S3 **Heatmap of the mapped genes at the time of 2:00, 6:00, 10:00, 14:00, 18:00, and 22:00**.

Supplementary Figure S4 **Functional categories of the mapped genes at the time of 2:00, 6:00, 10:00, 14:00, 18:00, and 22:00**.

Supplementary Figure S5 **The alternative splicing events at the time of 2:00, 6:00, 10:00, 14:00, 18:00, and 22:00**.

Supplementary Figure S6 **qRT-PCR analysis of nine randomly selected differentially expressed genes. Values are mean ± *SD* from three biological replicates**.

Supplementary Figure S7 **The size distribution of unique sRNA sequences**.

Supplementary Figure S8 **Heatmap of the mature miRNAs at the time of 2:00, 6:00, 10:00, 14:00, 18:00, and 22:00**.

Supplementary Table S1 **Primer sequences used in qRT-PCR**.

Supplementary Table S2 **Reads of mRNA sequencing in rice shoots at different time points**.

Supplementary Table S3 **Mapped events of mRNA sequencing in rice shoots at different time points**.

Supplementary Table S4 **Reads of small RNA sequencing in rice shoots at different time points**.

Supplementary Table S5 **Number of mature miRNA in rice shoots at different time points**.

Supplementary Table S6 **Target genes of osa-miR1440b, osa-miR2876-5p, osa-miR1877, and osa-miR5799 predicted by psRNATarget**.
