# Supplementary figures and images for: A Natural Light/Dark Cycle Regulation of Carbon-Nitrogen Metabolism and Gene Expression in Rice Shoots
Source: Front Plant Sci. 2016 Aug 30;7:1318. doi: 10.3389/fpls.2016.01318 (PMC5003941; doi:10.3389/fpls.2016.01318)

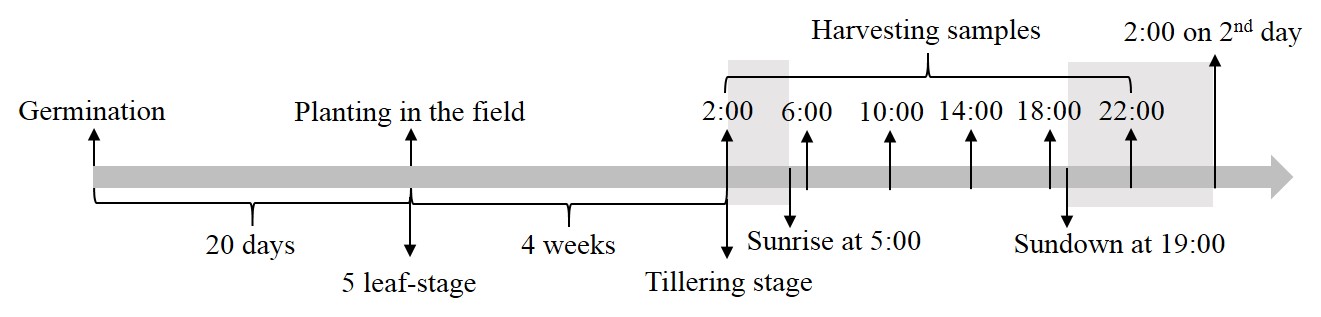

Supplement: Supplementary Figure S1 — The diagram of rice germination, planting, and harvesting. [file Image1.JPEG]

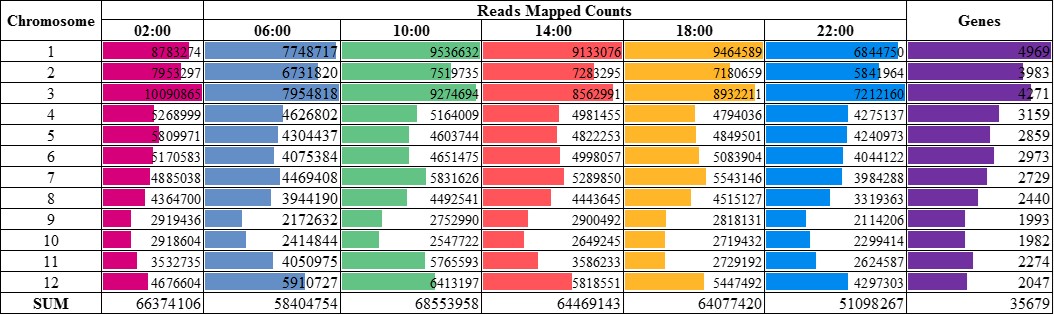

Supplement: Supplementary Figure S2 — The reads mapped to individual chromosome at the time of 2:00, 6:00, 10:00, 14:00, 18:00, and 22:00. [file Image2.JPEG]

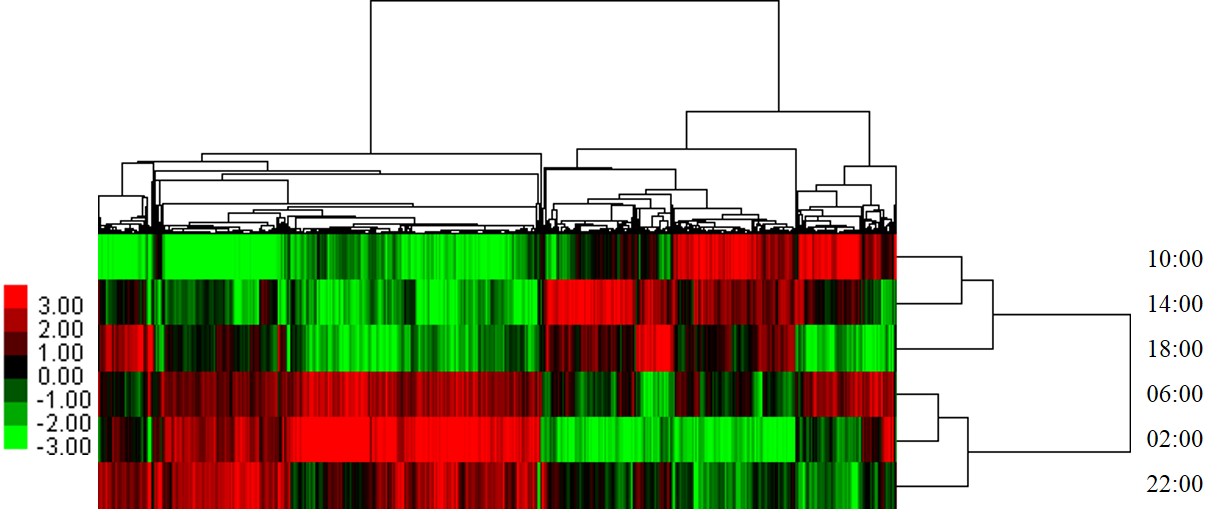

Supplement: Supplementary Figure S3 — Heatmap of the mapped genes at the time of 2:00, 6:00, 10:00, 14:00, 18:00, and 22:00. [file Image3.JPEG]

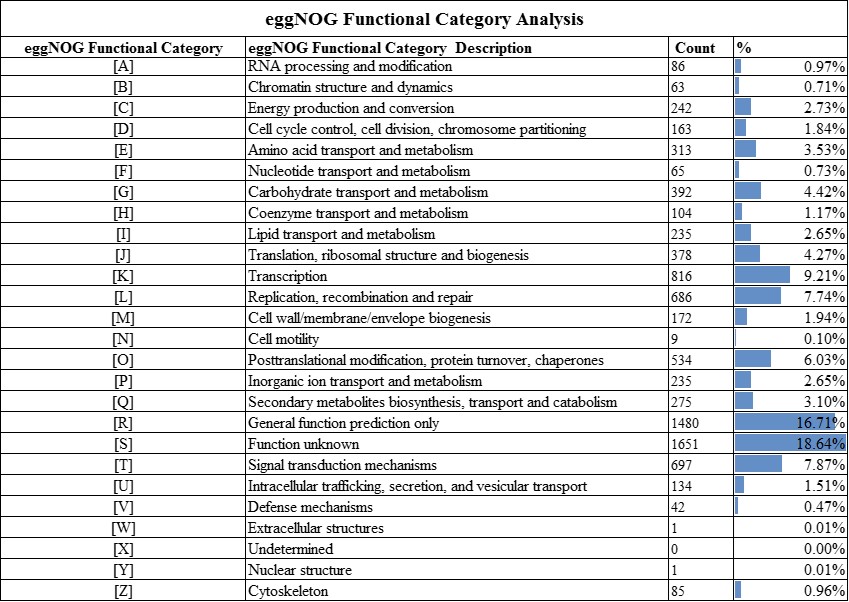

Supplement: Supplementary Figure S4 — Functional categories of the mapped genes at the time of 2:00, 6:00, 10:00, 14:00, 18:00, and 22:00. [file Image4.JPEG]

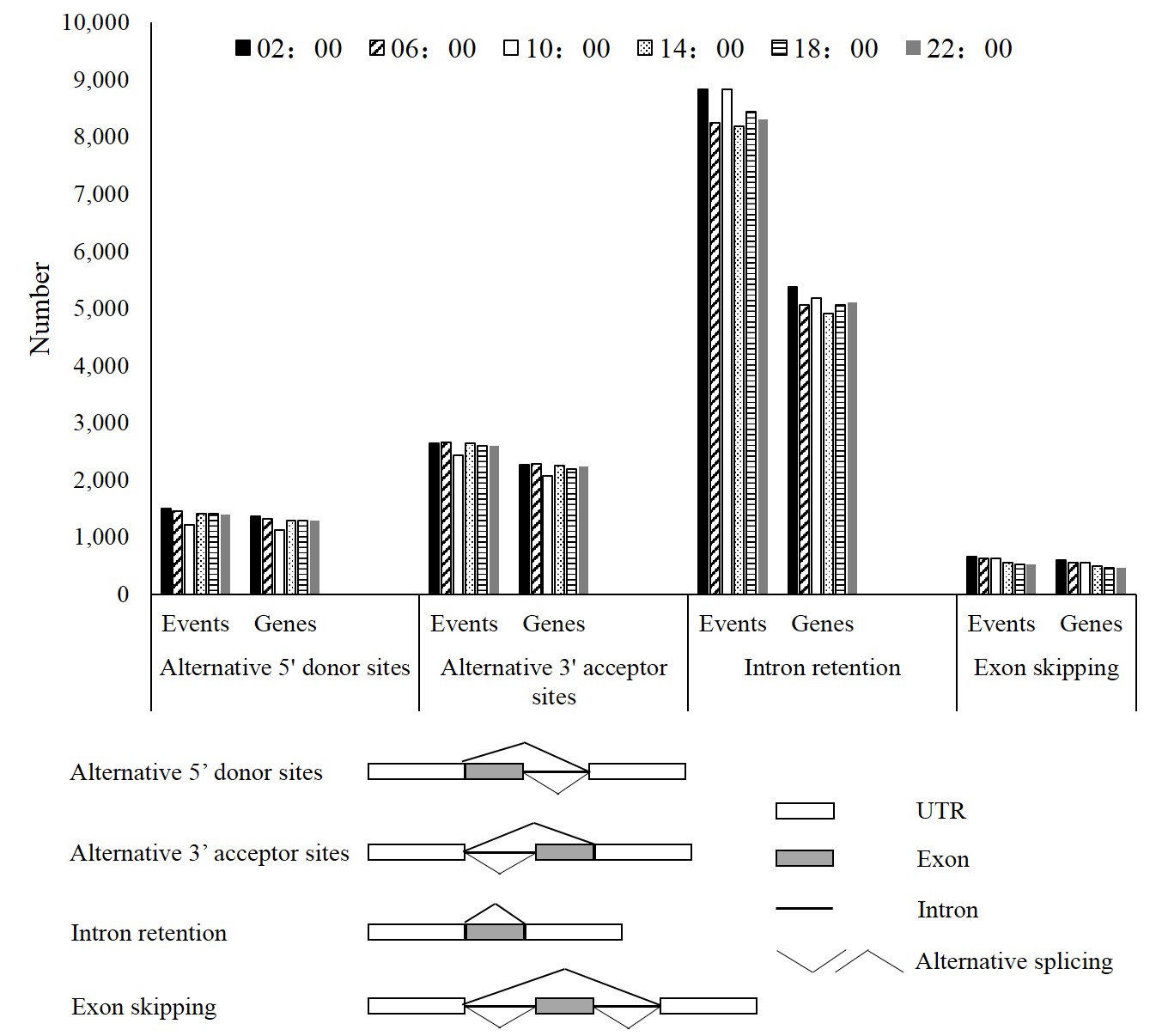

Supplement: Supplementary Figure S5 — The alternative splicing events at the time of 2:00, 6:00, 10:00, 14:00, 18:00, and 22:00. [file Image5.JPEG]

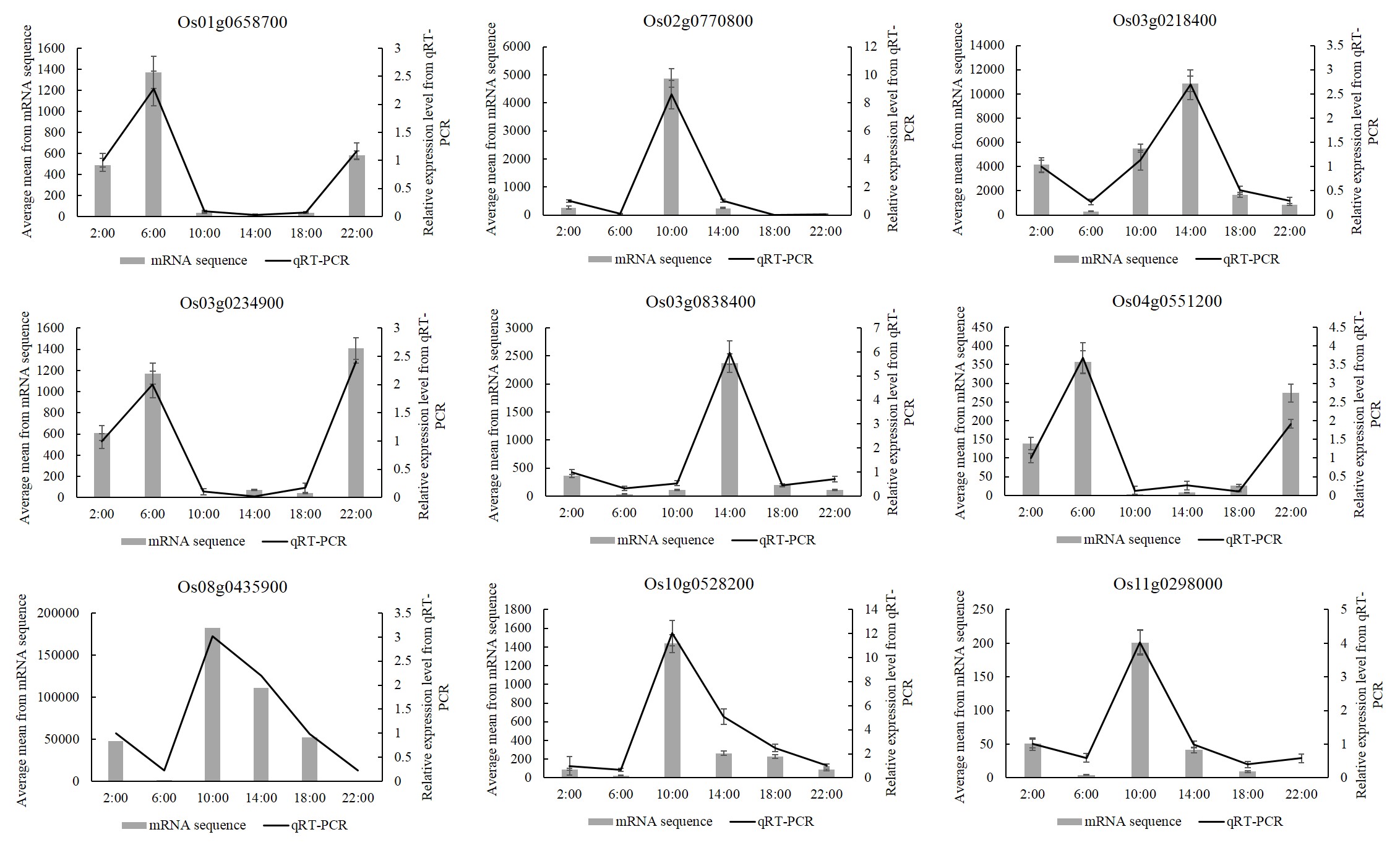

Supplement: Supplementary Figure S6 — qRT-PCR analysis of nine randomly selected differentially expressed genes. Values are mean ± SD from three biological replicates. [file Image6.JPEG]

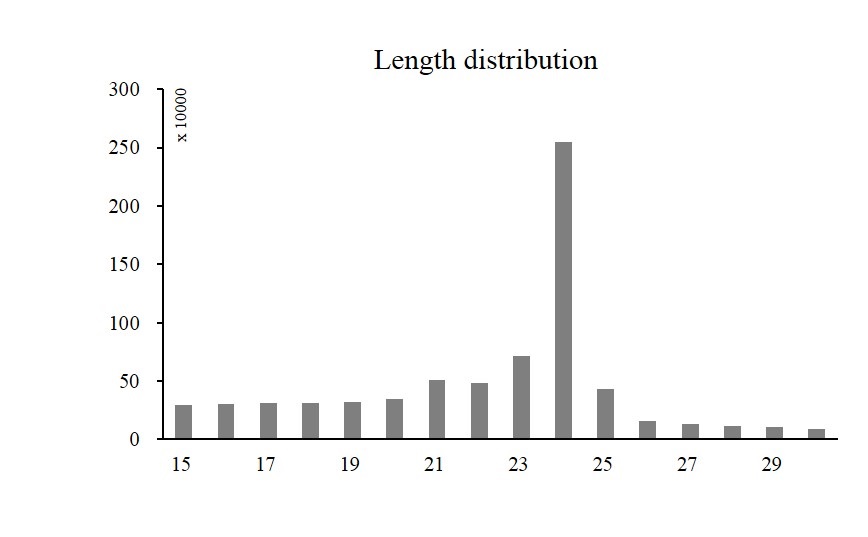

Supplement: Supplementary Figure S7 — The size distribution of unique sRNA sequences. [file Image7.JPEG]

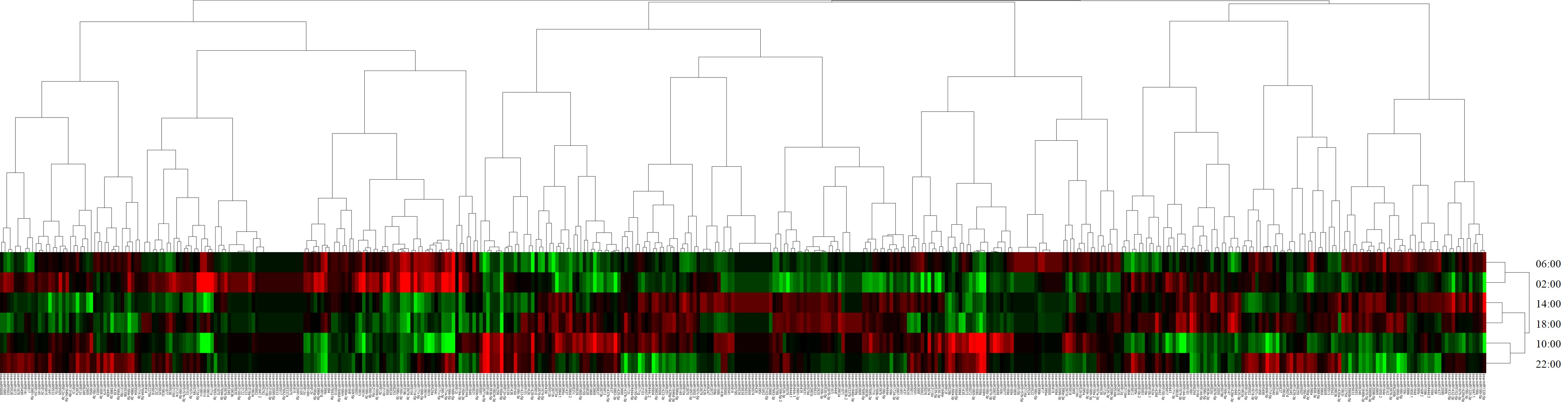

Supplement: Supplementary Figure S8 — Heatmap of the mature miRNAs at the time of 2:00, 6:00, 10:00, 14:00, 18:00, and 22:00. [file Image8.JPEG]
