# Supplementary material for: A Natural Light/Dark Cycle Regulation of Carbon-Nitrogen Metabolism and Gene Expression in Rice Shoots
Source: Front Plant Sci. 2016 Aug 30;7:1318. doi: 10.3389/fpls.2016.01318 (PMC5003941; doi:10.3389/fpls.2016.01318)
Supplement: Supplementary Table S1 — Primer sequences used in qRT-PCR. [file Table1.DOC]

**Supplementary Table S1**: Primer sequences used in qRT-PCR.

| Gene ID number | Primer sequence |
| --- | --- |
| Os01g0658700 | F: CCAGCAGCAAGGCTATCTTC |
|  | R: CCTGTTCCTTGCAAATCCAT |
| Os02g0770800 | F: GTCCATCGTTGAGAGCCCTA |
|  | R: GGCTCTTCTTGTCCATGAGC |
| Os03g0218400 | F: GAAGAAGAAGCACGAGGACA |
|  | R: GTGTAGGAGGCGAAGAAGGT |
| Os03g0234900 | F: CAAGATCTCCGTCGAACTGT |
|  | R: GAAGGGTGGTGAGGTTGTC |
| Os03g0838400 | F: ACATACTGTTCACGCTGACG |
|  | R: GTGCAGATGTTGGTGTTGAG |
| Os04g0551200 | F: GATATGGCTGAGGGAGGAGT |
|  | R: GGATCCAGTCTCTCATGTGG |
| Os08g0435900 | F: CTACCTTCCTAGGGCAATCC |
|  | R: TTGAGGTAGGTGGGAGAAGG |
| Os10g0528200 | F: GAGGACCTCAGCAACAAGAG |
|  | R: TCGATGTACTGCACGATGAC |
| Os11g0298000 | F: TCCAAGACGTACCAGGACAT |
|  | R: CTGAGGGAGATGGTGTAGGA |
| AK070531(ACTIN) | F: GACAATGGAACCGGAATGGTC |
|  | R: CCCAACCATAACGCCTGTATGT |
