# Supplementary material for: A Natural Light/Dark Cycle Regulation of Carbon-Nitrogen Metabolism and Gene Expression in Rice Shoots
Source: Front Plant Sci. 2016 Aug 30;7:1318. doi: 10.3389/fpls.2016.01318 (PMC5003941; doi:10.3389/fpls.2016.01318)
Supplement: Supplementary Table S2 — Reads of mRNA sequencing in rice shoots at different time points. [file Table2.DOCX]

**Supplementary Table S2 Reads of mRNA sequencing in rice shoots at different time points.**

| **Sample** | **Totalreads** | **Mapped reads** | **Multiple mapped reads** | **Uniquely mapped reads** |
| --- | --- | --- | --- | --- |
| 02:00 | 69,027,986 | 67,932,444(98.41%) | 1,802,473(2.65%)  1,516,096(2.54%)  2,279,427(3.23%)  2,021,058(3.05%)  1,674,754(2.56%)  9,475,165(15.69%) | 66,129,971(97.35%)  58,175,486(97.46%)  68,295,593(96.77%)  64,227,309(96.95%)  63,833,572(97.44%)  50,927,175(84.31%) |
| 06:00 | 60,990,646 | 59,691,582(97.87%) |  |  |
| 10:00 | 71,531,044 | 70,575,020(98.66%)  66,248,367(98.16%)  65,508,326(98.23%)  60,402,340(98.30%) |  |  |
| 14:00 | 67,490,066 |  |  |  |
| 18:00 | 66,686,142 |  |  |  |
| 22:00 | 61,447,438 |  |  |  |
