# Supplementary material for: A Natural Light/Dark Cycle Regulation of Carbon-Nitrogen Metabolism and Gene Expression in Rice Shoots
Source: Front Plant Sci. 2016 Aug 30;7:1318. doi: 10.3389/fpls.2016.01318 (PMC5003941; doi:10.3389/fpls.2016.01318)
Supplement: Supplementary Table S3 — Mapped events of mRNA sequencing in rice shoots at different time points. [file Table3.DOCX]

**Supplementary Table S3 Mapped events of mRNA sequencing in rice shoots at different time points.**

| **Sample** | **Mapped events** | **Reads mapped to genes** | **Reads mapped to intergene** | **Reads mapped to exon** |
| --- | --- | --- | --- | --- |
| 02:00 | 73,320,676 | 67,377,669 (91.89%)  59,793,582 (92.31%)  72,891,722 (90.84%)  67,937,305 (90.85%)  64,630,095 (90.96%)  88,222,684 (70.20%) | 5,943,007 (8.11%)  4,980,692 (7.69%)  7,348,639 (9.16%)  6,841,937 (9.15%)  6,424,125 (9.04%)  37,444,680 (29.80%) | 65,304,249 (96.92%)  58,040,045 (97.07%)  70,353,987 (96.52%)  65,977,997 (97.12%)  62,527,914 (96.75%)  86,521,167 (98.07%) |
| 06:00 | 64,774,274 |  |  |  |
| 10:00 | 80,240,361 |  |  |  |
| 14:00 | 74,779,242 |  |  |  |
| 18:00 | 71,054,220 |  |  |  |
| 22:00 | 125,667,364 |  |  |  |
