# Supplementary material for: A Natural Light/Dark Cycle Regulation of Carbon-Nitrogen Metabolism and Gene Expression in Rice Shoots
Source: Front Plant Sci. 2016 Aug 30;7:1318. doi: 10.3389/fpls.2016.01318 (PMC5003941; doi:10.3389/fpls.2016.01318)
Supplement: Supplementary Table S4 — Reads of small RNA sequencing in rice shoots at different time points. [file Table4.DOCX]

**Supplementary Table S4 Reads of small RNA sequencing in rice shoots at different time points.**

| **Sample** | **Raw reads** | **Clean reads** | **Clean reads (15-30 nt）** |
| --- | --- | --- | --- |
| 2:00 | 21,326,151 | 8,951,728 | 8,029,379 |
| 6:00 | 24,452,908 | 6,562,279 | 5,899,536 |
| 10:00 | 15,032,659 | 5,820,384 | 4,961,766 |
| 14:00 | 25,192,012 | 9,949,838 | 8,450,727 |
| 18:00 | 20,928,514 | 6,497,610 | 6,446,518 |
| 22:00 | 11,172,021 | 5,321,826 | 4,916,144 |
