# Supplementary material for: A Natural Light/Dark Cycle Regulation of Carbon-Nitrogen Metabolism and Gene Expression in Rice Shoots
Source: Front Plant Sci. 2016 Aug 30;7:1318. doi: 10.3389/fpls.2016.01318 (PMC5003941; doi:10.3389/fpls.2016.01318)
Supplement: Supplementary Table S5 — Number of mature miRNA in rice shoots at different time points. [file Table5.DOCX]

**Supplementary Table S5 Number of mature miRNA in rice shoots at different time points.**

| **Sample** | **2:00** | **6:00** | **10:00** | **14:00** | **18:00** | **22:00** |
| --- | --- | --- | --- | --- | --- | --- |
| miRNAs | 378 | 338 | 307 | 352 | 345 | 318 |
| Unique sequences | 11,894 | 7,627 | 4,637 | 8,639 | 7,944 | 5,283 |
| Total sequences | 318,392 | 160,286 | 108,952 | 226,405 | 206,049 | 84,621 |
