# Supplementary material for: A Natural Light/Dark Cycle Regulation of Carbon-Nitrogen Metabolism and Gene Expression in Rice Shoots
Source: Front Plant Sci. 2016 Aug 30;7:1318. doi: 10.3389/fpls.2016.01318 (PMC5003941; doi:10.3389/fpls.2016.01318)
Supplement: Supplementary Table S6 — Target genes of osa-miR1440b, osa-miR2876-5p, osa-miR1877, and osa-miR5799 predicted by psRNATarget. [file Table6.DOCX]

**Supplementary Table S6 Target genes of osa-miR1440b, osa-miR2876-5p, osa-miR1877 and osa-miR5799 predicted by psRNATarget.**

| **Id** | **Replicated sequence** | **Target id** | **Target description** | [**Inhibition**](http://plantgrn.noble.org/psRNATarget/?dowhat=Help#validcleavageregion) |
| --- | --- | --- | --- | --- |
| osa-miR1440b | TTTGGGAGAGTGGCATTTGAG | [LOC_Os10g38040.1](http://plantgrn.noble.org/psRNATarget/getseq.do?sessionid=1442975802530161&source=target&seqID=LOC_Os10g38040.1\|12010.m06586\|cDNA) | lysM domain containing protein, expressed | Cleavage |
|  |  | [LOC_Os03g63150.1](http://plantgrn.noble.org/psRNATarget/getseq.do?sessionid=1442975802530161&source=target&seqID=LOC_Os03g63150.1\|12003.m11184\|cDNA) | powdery mildew resistance protein PM3b, putative, expressed | Cleavage |
|  |  | [LOC_Os03g63150.2](http://plantgrn.noble.org/psRNATarget/getseq.do?sessionid=1442975802530161&source=target&seqID=LOC_Os03g63150.2\|12003.m35535\|cDNA) | powdery mildew resistance protein PM3b, putative, expressed | Cleavage |
|  |  | [LOC_Os04g28780.1](http://plantgrn.noble.org/psRNATarget/getseq.do?sessionid=1442975802530161&source=target&seqID=LOC_Os04g28780.1\|12004.m07992\|cDNA) | serine/threonine-protein kinase receptor precursor, putative, expressed | Cleavage |
|  |  | [LOC_Os03g51610.1](http://plantgrn.noble.org/psRNATarget/getseq.do?sessionid=1442975802530161&source=target&seqID=LOC_Os03g51610.1\|12003.m10139\|cDNA) | inositol-tetrakisphosphate 1-kinase 3, putative, expressed | Cleavage |
|  |  | [LOC_Os04g16771.1](http://plantgrn.noble.org/psRNATarget/getseq.do?sessionid=1442975802530161&source=target&seqID=LOC_Os04g16771.1\|12004.m101794\|cDNA) | chloroplast 50S ribosomal protein, putative, expressed | Cleavage |
|  |  | [LOC_Os04g16780.1](http://plantgrn.noble.org/psRNATarget/getseq.do?sessionid=1442975802530161&source=target&seqID=LOC_Os04g16780.1\|12004.m35074\|cDNA) | chloroplast 30S ribosomal protein S3, putative, expressed | Cleavage |
|  |  | [LOC_Os09g07460.1](http://plantgrn.noble.org/psRNATarget/getseq.do?sessionid=1442975802530161&source=target&seqID=LOC_Os09g07460.1\|12009.m04015\|cDNA) | tip elongation aberrant protein 1, putative, expressed | Translation |
|  |  | [LOC_Os03g48840.1](http://plantgrn.noble.org/psRNATarget/getseq.do?sessionid=1442975802530161&source=target&seqID=LOC_Os03g48840.1\|12003.m09887\|cDNA) | structural constituent of ribosome, putative, expressed | Translation |
|  |  | [LOC_Os03g44810.3](http://plantgrn.noble.org/psRNATarget/getseq.do?sessionid=1442975802530161&source=target&seqID=LOC_Os03g44810.3\|12003.m101424\|cDNA) | expressed protein | Translation |
|  |  | [LOC_Os03g44810.1](http://plantgrn.noble.org/psRNATarget/getseq.do?sessionid=1442975802530161&source=target&seqID=LOC_Os03g44810.1\|12003.m09516\|cDNA) | expressed protein | Translation |
|  |  | [LOC_Os03g44810.2](http://plantgrn.noble.org/psRNATarget/getseq.do?sessionid=1442975802530161&source=target&seqID=LOC_Os03g44810.2\|12003.m101423\|cDNA) | expressed protein | Translation |
|  |  | [LOC_Os09g18260.1](http://plantgrn.noble.org/psRNATarget/getseq.do?sessionid=1442975802530161&source=target&seqID=LOC_Os09g18260.1\|12009.m05084\|cDNA) | senescence-induced receptor-like serine/threonine-protein kinase precursor, putative | Cleavage |
| osa-miR2876-5p | AATTGCTGGCAGCACTGTTTA | [LOC_Os02g39010.4](http://plantgrn.noble.org/psRNATarget/getseq.do?sessionid=1442976223706847&source=target&seqID=LOC_Os02g39010.4\|12002.m34019\|cDNA) | PITSLRE serine/threonine-protein kinase CDC2L1, putative, expressed | Cleavage |
|  |  | [LOC_Os02g39330.1](http://plantgrn.noble.org/psRNATarget/getseq.do?sessionid=1442976223706847&source=target&seqID=LOC_Os02g39330.1\|12002.m09021\|cDNA) | endochitinase PR4 precursor, putative, expressed | Cleavage |
|  |  | [LOC_Os12g06920.1](http://plantgrn.noble.org/psRNATarget/getseq.do?sessionid=1442976223706847&source=target&seqID=LOC_Os12g06920.1\|12012.m04682\|cDNA) | NBS-LRR disease resistance protein, putative | Cleavage |
|  |  | [LOC_Os01g42950.2](http://plantgrn.noble.org/psRNATarget/getseq.do?sessionid=1442976223706847&source=target&seqID=LOC_Os01g42950.2\|12001.m42677\|cDNA) | ATP binding protein, putative, expressed | Cleavage |
|  |  | [LOC_Os01g42950.1](http://plantgrn.noble.org/psRNATarget/getseq.do?sessionid=1442976223706847&source=target&seqID=LOC_Os01g42950.1\|12001.m10558\|cDNA) | ATP binding protein, putative, expressed | Cleavage |
|  |  | [LOC_Os07g12540.1](http://plantgrn.noble.org/psRNATarget/getseq.do?sessionid=1442976223706847&source=target&seqID=LOC_Os07g12540.1\|12007.m05706\|cDNA) | expressed protein | Cleavage |
|  |  | [LOC_Os03g21520.1](http://plantgrn.noble.org/psRNATarget/getseq.do?sessionid=1442976223706847&source=target&seqID=LOC_Os03g21520.1\|12003.m07556\|cDNA) | expressed protein | Cleavage |
|  |  | [LOC_Os03g21520.2](http://plantgrn.noble.org/psRNATarget/getseq.do?sessionid=1442976223706847&source=target&seqID=LOC_Os03g21520.2\|12003.m101332\|cDNA) | expressed protein | Cleavage |
| osa-miR1877 | AGATGACATGTAAACAATGAGGGG | [LOC_Os09g39360.1](http://plantgrn.noble.org/psRNATarget/getseq.do?sessionid=1442976247332607&source=target&seqID=LOC_Os09g39360.1\|12009.m21958\|cDNA) | expressed protein | Cleavage |
|  |  | [LOC_Os01g73170.1](http://plantgrn.noble.org/psRNATarget/getseq.do?sessionid=1442976247332607&source=target&seqID=LOC_Os01g73170.1\|12001.m13346\|cDNA) | peroxidase 12 precursor, putative, expressed | Cleavage |
|  |  | [LOC_Os01g72670.4](http://plantgrn.noble.org/psRNATarget/getseq.do?sessionid=1442976247332607&source=target&seqID=LOC_Os01g72670.4\|12001.m43384\|cDNA) | expressed protein | Cleavage |
|  |  | [LOC_Os05g45880.1](http://plantgrn.noble.org/psRNATarget/getseq.do?sessionid=1442976247332607&source=target&seqID=LOC_Os05g45880.1\|12005.m08713\|cDNA) | ethanolamine kinase 1, putative, expressed | Cleavage |
|  |  | [LOC_Os09g25980.1](http://plantgrn.noble.org/psRNATarget/getseq.do?sessionid=1442976247332607&source=target&seqID=LOC_Os09g25980.1\|12009.m05750\|cDNA) | glutamate receptor 2.6 precursor, putative | Cleavage |
|  |  | [LOC_Os08g14360.1](http://plantgrn.noble.org/psRNATarget/getseq.do?sessionid=1442976247332607&source=target&seqID=LOC_Os08g14360.1\|12008.m05560\|cDNA) | expressed protein | Translation |
|  |  | [LOC_Os12g19304.4](http://plantgrn.noble.org/psRNATarget/getseq.do?sessionid=1442976247332607&source=target&seqID=LOC_Os12g19304.4\|12012.m56488\|cDNA) | quinolinate synthetase A protein, expressed | Cleavage |
|  |  | [LOC_Os12g19304.2](http://plantgrn.noble.org/psRNATarget/getseq.do?sessionid=1442976247332607&source=target&seqID=LOC_Os12g19304.2\|12012.m56487\|cDNA) | quinolinate synthetase A protein, expressed | Cleavage |
|  |  | [LOC_Os12g19304.1](http://plantgrn.noble.org/psRNATarget/getseq.do?sessionid=1442976247332607&source=target&seqID=LOC_Os12g19304.1\|12012.m05882\|cDNA) | quinolinate synthetase A protein, expressed | Cleavage |
|  |  | [LOC_Os12g19304.3](http://plantgrn.noble.org/psRNATarget/getseq.do?sessionid=1442976247332607&source=target&seqID=LOC_Os12g19304.3\|12012.m56486\|cDNA) | quinolinate synthetase A protein, expressed | Cleavage |
|  |  | [LOC_Os03g63770.3](http://plantgrn.noble.org/psRNATarget/getseq.do?sessionid=1442976247332607&source=target&seqID=LOC_Os03g63770.3\|12003.m35540\|cDNA) | RCD1, putative, expressed | Cleavage |
|  |  | [LOC_Os03g63770.5](http://plantgrn.noble.org/psRNATarget/getseq.do?sessionid=1442976247332607&source=target&seqID=LOC_Os03g63770.5\|12003.m35646\|cDNA) | RCD1, putative, expressed | Cleavage |
|  |  | [LOC_Os03g63770.2](http://plantgrn.noble.org/psRNATarget/getseq.do?sessionid=1442976247332607&source=target&seqID=LOC_Os03g63770.2\|12003.m34959\|cDNA) | RCD1, putative, expressed | Cleavage |
|  |  | [LOC_Os01g48550.1](http://plantgrn.noble.org/psRNATarget/getseq.do?sessionid=1442976247332607&source=target&seqID=LOC_Os01g48550.1\|12001.m11045\|cDNA) | expressed protein | Cleavage |
|  |  | [LOC_Os01g47350.1](http://plantgrn.noble.org/psRNATarget/getseq.do?sessionid=1442976247332607&source=target&seqID=LOC_Os01g47350.1\|12001.m10930\|cDNA) | naphthoate synthase, putative, expressed | Cleavage |
|  |  | [LOC_Os03g31230.1](http://plantgrn.noble.org/psRNATarget/getseq.do?sessionid=1442976247332607&source=target&seqID=LOC_Os03g31230.1\|12003.m08371\|cDNA) | DNA binding protein, putative, expressed | Translation |
|  |  | [LOC_Os03g31230.2](http://plantgrn.noble.org/psRNATarget/getseq.do?sessionid=1442976247332607&source=target&seqID=LOC_Os03g31230.2\|12003.m35283\|cDNA) | DNA binding protein, putative, expressed | Translation |
|  |  | [LOC_Os02g24330.2](http://plantgrn.noble.org/psRNATarget/getseq.do?sessionid=1442976247332607&source=target&seqID=LOC_Os02g24330.2\|12002.m07580\|cDNA) | translation initiation factor, putative, expressed | Cleavage |
|  |  | [LOC_Os01g58750.1](http://plantgrn.noble.org/psRNATarget/getseq.do?sessionid=1442976247332607&source=target&seqID=LOC_Os01g58750.1\|12001.m12017\|cDNA) | expressed protein | Translation |
| osa-miR5799 | AGACGAATGGTCAAACGTTGGACA | [LOC_Os11g34910.1](http://plantgrn.noble.org/psRNATarget/getseq.do?sessionid=1442976256348358&source=target&seqID=LOC_Os11g34910.1\|12011.m28631\|cDNA) | expressed protein | Cleavage |
|  |  | [LOC_Os09g33710.1](http://plantgrn.noble.org/psRNATarget/getseq.do?sessionid=1442976256348358&source=target&seqID=LOC_Os09g33710.1\|12009.m06420\|cDNA) | beta-glucosidase homolog precursor, putative, expressed | Cleavage |
|  |  | [LOC_Os04g58730.2](http://plantgrn.noble.org/psRNATarget/getseq.do?sessionid=1442976256348358&source=target&seqID=LOC_Os04g58730.2\|12004.m35526\|cDNA) | DNA binding protein, putative, expressed | Cleavage |
|  |  | [LOC_Os04g58730.1](http://plantgrn.noble.org/psRNATarget/getseq.do?sessionid=1442976256348358&source=target&seqID=LOC_Os04g58730.1\|12004.m10753\|cDNA) | DNA binding protein, putative, expressed | Cleavage |
|  |  | [LOC_Os09g37540.1](http://plantgrn.noble.org/psRNATarget/getseq.do?sessionid=1442976256348358&source=target&seqID=LOC_Os09g37540.1\|12009.m06701\|cDNA) | carboxy-lyase, putative, expressed | Cleavage |
|  |  | [LOC_Os10g28690.1](http://plantgrn.noble.org/psRNATarget/getseq.do?sessionid=1442976256348358&source=target&seqID=LOC_Os10g28690.1\|12010.m05747\|cDNA) | expressed protein | Cleavage |
|  |  | [LOC_Os03g45990.1](http://plantgrn.noble.org/psRNATarget/getseq.do?sessionid=1442976256348358&source=target&seqID=LOC_Os03g45990.1\|12003.m09620\|cDNA) | expressed protein | Cleavage |
|  |  | [LOC_Os11g09260.1](http://plantgrn.noble.org/psRNATarget/getseq.do?sessionid=1442976256348358&source=target&seqID=LOC_Os11g09260.1\|12011.m05123\|cDNA) | expressed protein | Cleavage |
|  |  | [LOC_Os01g56720.1](http://plantgrn.noble.org/psRNATarget/getseq.do?sessionid=1442976256348358&source=target&seqID=LOC_Os01g56720.1\|12001.m11829\|cDNA) | expressed protein | Cleavage |
|  |  | [LOC_Os05g39540.1](http://plantgrn.noble.org/psRNATarget/getseq.do?sessionid=1442976256348358&source=target&seqID=LOC_Os05g39540.1\|12005.m08132\|cDNA) | zinc transporter 4, chloroplast precursor, putative, expressed | Cleavage |
|  |  | [LOC_Os06g37300.1](http://plantgrn.noble.org/psRNATarget/getseq.do?sessionid=1442976256348358&source=target&seqID=LOC_Os06g37300.1\|12006.m08265\|cDNA) | ent-kaurene oxidase, putative, expressed | Cleavage |
|  |  | [LOC_Os07g03377.1](http://plantgrn.noble.org/psRNATarget/getseq.do?sessionid=1442976256348358&source=target&seqID=LOC_Os07g03377.1\|12007.m04809\|cDNA) | pathogenesis-related protein 1 precursor, putative, expressed | Cleavage |
|  |  | [LOC_Os04g33780.3](http://plantgrn.noble.org/psRNATarget/getseq.do?sessionid=1442976256348358&source=target&seqID=LOC_Os04g33780.3\|12004.m35571\|cDNA) | cysteine-type peptidase, putative, expressed | Cleavage |
|  |  | [LOC_Os04g33780.2](http://plantgrn.noble.org/psRNATarget/getseq.do?sessionid=1442976256348358&source=target&seqID=LOC_Os04g33780.2\|12004.m35308\|cDNA) | cysteine-type peptidase, putative, expressed | Cleavage |
|  |  | [LOC_Os04g33780.1](http://plantgrn.noble.org/psRNATarget/getseq.do?sessionid=1442976256348358&source=target&seqID=LOC_Os04g33780.1\|12004.m08473\|cDNA) | cysteine-type peptidase, putative, expressed | Cleavage |
|  |  | [LOC_Os04g09900.2](http://plantgrn.noble.org/psRNATarget/getseq.do?sessionid=1442976256348358&source=target&seqID=LOC_Os04g09900.2\|12004.m35117\|cDNA) | ent-kaurene synthase A, chloroplast precursor, putative, expressed | Cleavage |
|  |  | [LOC_Os04g33720.1](http://plantgrn.noble.org/psRNATarget/getseq.do?sessionid=1442976256348358&source=target&seqID=LOC_Os04g33720.1\|12004.m08467\|cDNA) | beta-fructofuranosidase, insoluble isoenzyme 3 precursor, putative, expressed | Cleavage |
|  |  | [LOC_Os04g20474.3](http://plantgrn.noble.org/psRNATarget/getseq.do?sessionid=1442976256348358&source=target&seqID=LOC_Os04g20474.3\|12004.m101480\|cDNA) | UDP-glycosyltransferase/ transferase, transferring glycosyl groups, putative, expressed | Cleavage |
|  |  | [LOC_Os04g20474.2](http://plantgrn.noble.org/psRNATarget/getseq.do?sessionid=1442976256348358&source=target&seqID=LOC_Os04g20474.2\|12004.m78946\|cDNA) | UDP-glycosyltransferase/ transferase, transferring glycosyl groups, putative, expressed | Cleavage |
|  |  | [LOC_Os04g20474.1](http://plantgrn.noble.org/psRNATarget/getseq.do?sessionid=1442976256348358&source=target&seqID=LOC_Os04g20474.1\|12004.m07204\|cDNA) | UDP-glycosyltransferase/ transferase, transferring glycosyl groups, putative, expressed | Cleavage |
|  |  | [LOC_Os05g09660.1](http://plantgrn.noble.org/psRNATarget/getseq.do?sessionid=1442976256348358&source=target&seqID=LOC_Os05g09660.1\|12005.m05479\|cDNA) | HAD superfamily phosphatase containing protein, expressed | Cleavage |
|  |  | [LOC_Os11g37540.1](http://plantgrn.noble.org/psRNATarget/getseq.do?sessionid=1442976256348358&source=target&seqID=LOC_Os11g37540.1\|12011.m07599\|cDNA) | protein phosphatase 2C containing protein, expressed | Cleavage |
|  |  | [LOC_Os09g20490.1](http://plantgrn.noble.org/psRNATarget/getseq.do?sessionid=1442976256348358&source=target&seqID=LOC_Os09g20490.1\|12009.m05256\|cDNA) | carbohydrate transporter/ sugar porter/ transporter, putative, expressed | Cleavage |
|  |  | [LOC_Os02g14290.1](http://plantgrn.noble.org/psRNATarget/getseq.do?sessionid=1442976256348358&source=target&seqID=LOC_Os02g14290.1\|12002.m100155\|cDNA) | scramblase family protein, expressed | Cleavage |
|  |  | [LOC_Os12g01680.1](http://plantgrn.noble.org/psRNATarget/getseq.do?sessionid=1442976256348358&source=target&seqID=LOC_Os12g01680.1\|12012.m04171\|cDNA) | macrophage migration inhibitory factor, putative, expressed | Cleavage |
|  |  | [LOC_Os05g11560.2](http://plantgrn.noble.org/psRNATarget/getseq.do?sessionid=1442976256348358&source=target&seqID=LOC_Os05g11560.2\|12005.m27739\|cDNA) | aquaporin NIP-type, putative, expressed | Cleavage |
|  |  | [LOC_Os04g37820.1](http://plantgrn.noble.org/psRNATarget/getseq.do?sessionid=1442976256348358&source=target&seqID=LOC_Os04g37820.1\|12004.m08771\|cDNA) | cytokinin-O-glucosyltransferase 2, putative, expressed | Cleavage |
|  |  | [LOC_Os09g07320.2](http://plantgrn.noble.org/psRNATarget/getseq.do?sessionid=1442976256348358&source=target&seqID=LOC_Os09g07320.2\|12009.m21970\|cDNA) | ethanol tolerance protein GEKO1, putative, expressed | Cleavage |
|  |  | [LOC_Os06g11500.1](http://plantgrn.noble.org/psRNATarget/getseq.do?sessionid=1442976256348358&source=target&seqID=LOC_Os06g11500.1\|12006.m05867\|cDNA) | minichromosome maintenance protein MCM, putative, expressed | Cleavage |
|  |  | [LOC_Os12g38210.2](http://plantgrn.noble.org/psRNATarget/getseq.do?sessionid=1442976256348358&source=target&seqID=LOC_Os12g38210.2\|12012.m26937\|cDNA) | spotted leaf protein 11, putative, expressed | Cleavage |
